# Protective Effects of Resveratrol Against Perfluorooctanoic Acid-Induced Testicular and Epididymal Toxicity in Adult Rats Exposed During Their Prepubertal Period

**DOI:** 10.3390/toxics13020111

**Published:** 2025-01-29

**Authors:** R. Pavani, K. Venkaiah, P. Gnana Prakasam, Vijaya R. Dirisala, P. Gopi Krishna, B. Kishori, S. B. Sainath

**Affiliations:** 1 Department of Biotechnology, Vikrama Simhapuri University, Nellore 524324, India; pavani.pearl1991@gmail.com (R.P.); venkatkaduru13@gmail.com (K.V.); gnanaprakashbio@gmail.com (P.G.P.); 2 Department of Biotechnology, Vignan’s Foundation for Science, Technology and Research, Vadlamudi, Guntur 522213, India; drdirisala@gmail.com; 3 Department of Zoology, Vikrama Simhapuri University PG Centre, Kavali 524201, India; pitchika.gopikrishna@gmail.com; 4 Department of Biotechnology, Sri Padmavathi Mahila Viswavidyalayam, Tirupati 517502, India; kktinku@rediffmail.com; 5 Department of Food Technology, Vikrama Simhapuri University, Nellore 524324, India

**Keywords:** antioxidants, endocrine disruption, perfluorooctanoic acid, rats, sperm, testosterone, transcriptomics

## Abstract

The antioxidant properties of resveratrol (RES) against oxidative toxicity induced by testicular toxicants are well documented. The current study aimed to investigate the probable beneficial role of RES on male reproduction in adult rats following prepubertal exposure to perfluorooctanoic acid (PFOA). Healthy rats of the Wistar strain (23 days old) were allocated into four groups. Rats in group I did not receive any treatment, while rats in groups II, III, and IV received RES, PFOA, and RES + PFOA, respectively, between days 23 and 56 and were monitored for up to 90 days. Exposure to PFOA resulted in a significant reduction in spermiogram parameters, testicular 3β- and 17β-HSD activity levels, and circulatory levels of testosterone. A significant elevation in LPx, PCs, H_2_O_2_, and O_2_^−^, associated with a concomitant reduction in SOD, CAT, GPx, GR, and GSH, was noticed in the testes, as well as region-specific changes in pro- and antioxidants in the epididymides of exposed rats compared to controls. A significant increase in serum FSH and LH, testicular cholesterol levels, and caspase-3 activity was observed in PFOA-exposed rats compared to controls. Histological analysis revealed that the integrity of the testes was deteriorated in PFOA-exposed rats. Transcriptomic profiling of the testes and epididymides revealed 98 and 611 altered genes, respectively. In the testes, apoptosis and glutathione pathways were disrupted, while in the epididymides, glutathione and bile secretion pathways were altered in PFOA-exposed rats. PFOA exposure resulted in the down-regulation in the testes of *17β-HSD, StAR, nfe2l2, ar, Lhcgr,* and mRNA levels, associated with the up-regulation of *casp3* mRNA, and down-regulation of *alpha 1 adrenoceptor*, *muscarinic choline receptor 3*, and *androgen receptor* in the epididymides of exposed rats compared to the controls. These events might lead to male infertility in PFOA-exposed rats. In contrast, restoration of selected reproductive variables was observed in RES plus PFOA-exposed rats compared to rats exposed to PFOA alone. Taken together, we postulate that prepubertal exposure to PFOA triggered oxidative damage and altered genes in the testes and epididymides, leading to suppressed male reproductive health in adult rats, while RES, with its steroidogenic, antiapoptotic, and antioxidant effects, restored PFOA-induced fertility potential in rats.

## 1. Introduction

The deterioration of male reproductive health, as indicated by testicular cancer, the inhibition of testosterone, and reduced sperm counts among the population at reproductive age, is alarming. One of the plausible factors linked to this reduced fertility efficacy is chemicals with endocrine-disrupting properties [1]. Perfluoro- and polyfluoroalkyl substances (PFASs) are types of endocrine disruptors, with different lengths of carbon chains, functional groups, and fluorination degrees [2]. Perfluorooctanoic acid (PFOA) is a type of PFAS which is used as a surfactant in the manufacture of nonstick cookware, firefighting foams, food packaging, and outdoor clothes, carpets, and footwear [3]. Due to its wide usage and persistent nature (exhibiting a long half-life of approximately 92 years before its complete elimination from water [4]), PFOA can be found in drinking water, food, and the air [5,6]. Studies have shown that PFOA has been detected at a concentration of 0.02 to 0.35 ppb in potable water [7,8,9]. A survey conducted by the National Health and Nutrition Examination in 2017–2018 revealed that approximately 1.42 parts per billion (ppb) of PFOA were found in the serum of humans [10]. According to [11,12,13], PFOA has been categorized as a carcinogen. The first nationwide bio-monitoring survey on PFASs in the Indian population revealed that PFOA was one of the predominating PFASs [14]. Published studies have detected PFOA in biological samples including serum [15] and seminal plasma [16]. Human exposure to PFOA is alarming because of its persistent nature and, thus, the likelihood of it affecting multiple generations; therefore, a systematic study on the implications of legacy PFOA on male reproductive health is warranted.

Studies have shown that PFOA intoxication leads to hepatotoxicity [17], nephrotoxicity [18], neurotoxicity [19], reproductive toxicity [20], thyroid gland disruption [21], and pancreatic toxicity [22]. With regard to male reproduction, PFOA-induced reprotoxicity, at least in part, underlies deteriorated spermatogenesis and sperm quality, inhibition of testosterone levels, abnormal development of male reproductive organs, inhibition of Leydig cell development, and an increase in circulatory levels of estradiol [23,24,25,26]. Further, exposure to PFOA provokes testicular oxidative stress, as indicated by the excess production of free radicals and, in parallel, failure of the counter-attack mechanism of enzymatic and non-enzymatic antioxidants [27,28,29]. Most significant studies on male reproductive toxicity in response to PFOA exposure are related to adults, whereas studies pertaining to the effects of prepubertal exposure to PFOA on male reproductive health in adulthood are limited. The prepubertal period is the most crucial period in reproductive development, during which testosterone-dependent processes like the transformation of fetal Leydig cells to adult Leydig cells, Sertoli cell development, and epididymis development take place [30]. Therefore, it is conceivable that the exposure of males during the prepubertal period to endocrine-disrupting chemicals which interfere with testosterone biosynthesis and also induce oxidative stress may adversely affect the fertility potential of males later in life. This hypothesis was tested in this study using PFOA as the test chemical.

At present, using antioxidant therapy to protect against testicular toxicants is one of the most important areas of biomedicine. Resveratrol (RES; 3,4′,5-trihydroxystilbene) is an antioxidant present in more than 70 plants and can be found in products including grape wine, chocolates, peanuts, mulberries, strawberries, and herbal medicines [31,32,33]. The role of RES as an anti-aging, anti-asthma, anti-arthritis, anti-cancer, antioxidant, antitumor, anti-inflammatory, anti-diabetic, hepatoprotective, and neuroprotective agent is well known [34,35]. Previously, it has been shown that the supplementation of RES ameliorated testosterone levels, spermatogenesis, and the activity levels of 3β- and 17β-hydroxysteroid dehydrogenases, as well as restoring the testicular architecture in animal models against a range of chemicals, including doxorubicin, cisplatin, nitric oxide, benzo(a)pyrene, combination of streptozotocin-nicotinamide, 2,3,7,8-tetrachlorodibenzo-*p*-dioxin, malathion, nicotine, finasteride, and sulfoxaflor [36,37,38,39,40,41,42,43,44,45,46,47].

The precise regulation of genes and their interactions is key for proper spermatogenesis, testosterone biosynthesis, and sperm maturation events, and thus, it is conceivable that the PFOA induced deterioration of testicular and epididymal functions may entail perturbations at the molecular level. To test this notion, we employed transcriptomic analysis, a well acknowledged tool to understand the molecular basis of toxicants under different experimental conditions [48,49,50,51,52].

The central objective of this study was to determine whether prepubertal exposure to PFOA would affect the testicular and epididymal functions at adulthood and, if so, whether supplementation with resveratrol would result in reversal effects on PFOA induced reproductive toxicity in adult male rats.

## 2. Materials and Methods

### 2.1. Chemicals

Test chemicals used in this study, such as perfluorooctanoic acid (PFOA: CAS no. 335-67-1; Purity: 95%) were obtained from Sigma chemicals Co, St. Louis, MI, USA). Resveratrol (RES; CAS No. 501-36-0; Purity > 99%) was obtained from TCI chemicals (Chennai, Tamilnadu, India) Pvt. Ltd. Molecular kits and all other chemicals were procured from TaKaRa, Sigma Chemicals, and Merck (Bengaluru, Karnataka, India).

### 2.2. Procurement and Maintenance of Experimental Animals

Healthy Wistar strain albino rats (15 days old) were obtained from an authorized vendor (Bengaluru, Karnataka). All the animals were transported to the laboratory in an air-conditioned vehicle without causing any stress. During their acclimatization (~7 days) to the laboratory environment, they were maintained in standard polypropylene rat cages covered with paddy husk as the bedding material. The laboratory conditions were as follows: temperature of 22 to 25 °C, 12-h light and 12-dark cycle, and relative humanity of 50 ± 5 °C. Food and tap water were provided adequately i.e., ad libitum. The experiments in this study were carried out as per the regulations of Committee for the Purpose of Control and Supervision on Experiments on Animals, Government of India [53], and were approved by the Institutional Animal Ethical Committee (vide No. 1837/PO/ RcBiBt/S/15/CPCSEA).

### 2.3. Experimental Design

Rats at prepubertal age (23 days old; body weights: 35 g to 38 g) were randomly divided into four groups of ten rats each. In group I, rats did not receive any treatment and were considered as controls. Rats in group II, III, and IV served as experimental groups. Rats in group II were given RES (20 mg/Kg body weight/day) orally via gavage, while rats in group III were exposed to PFOA (20 mg/Kg body weight/day). In group IV, the rats were treated with both PFOA and RES at selected doses orally via gavage. The test chemicals were given to rats from day 23 to 56 and maintained up to 90th day (completion of one spermatogenic cycle). In the present study, the doses of test chemicals were selected based on previous studies. PFOA was selected at a dose of 20 mg/kg/day [23] and RES was 20 mg/kg body weight [37]. PFOA was dissolved in milliQ water and RES was dissolved in DMSO (0.2%). The timeline during the prepubertal period (23 days to 56 days) is a critical window period during which Leydig cell proliferation and differentiation in rats takes place, and moreover, the immature Leydig cells are completely transformed into adult Leydig cells. The maturation and development of the male reproductive tract in rats and the onset of puberty on day 45 are also covered in the prepubertal timeline [26,54,55]. At 90 days, rats from the control, PFOA exposed, RES treated, and PFOA + RES treated groups were analyzed for fertility studies, followed by assessing selected reproductive variables.

### 2.4. Fertility Studies

The rats from the control and experimental groups were cohabited with healthy virgin female rats at proestrus cycle (1:1 ratio) in order to assess fertility. The cohabitation period was set to four days. The female rats were introduced into the native cages of male rat at 6:00 p.m. The next day morning, vaginal smears were collected from the females using Pasteur pipettes containing physiological saline (0.9% NaCl) and microscopically inspected for the presence of sperm. These females were then considered gestation day 1 (GD1) and were separated and maintained in separate cages. The fertility efficacy parameters, like the number of copulation trials, mating and fertility index, pre- and post-implantation loss, and number of live fetuses, were determined. The methodology is described in the Supplementary Materials. Further, sexual behavior parameters, like number of mounts, number of intromissions until first ejaculation, latency to the first mount, first intromission, and first ejaculation, were also analyzed in control and experimental rats [56].

### 2.5. Collection of Blood and Necropsy

Male rats were fasted overnight after completion of the fertility examination and sacrificed humanely via cervical dislocation. The somatic organs like brain, kidney, and liver, and reproductive organs like testis and accessory sex organs were isolated and weighed to the nearest milligram (Shimadzu Model: BI-220H, Mumbai, India). Their relative weights were determined using the formula: tissue somatic index = weight of the tissue/weight of animal × 100. The collection of blood was done before necropsy through cardiac puncture using a heparinized syringe. Blood samples were kept overnight at 4 °C, followed by a centrifugation step at 2000 rpm for 15 min. to separate serum. The serum was stored at −20 °C until further hormonal analysis.

### 2.6. Spermiogram Analysis

The daily sperm count in the testis was determined in accordance with the methods described in [57,58]. Cauda epididymis was used to analyze sperm variables like motility, viability, tail coiling, and morphological changes. Sperm counts and progressive motility [59] and sperm viability [60] tests were performed according to standard protocols. The hypo-osmotic swelling test gives valuable information about the membrane integrity of sperm. The test is based on the principle that when intact sperm are exposed to hypo-osmotic solution, the tail coils, while sperm with membrane damage exhibit no tail coiling, as observed under a microscope [61]. Changes in the morphology of sperm were identified based on the protocol described by Linder et al. [62]. Alkaline comet assay was used to analyze sperm DNA integrity using the protocol followed by Miranda-Spooner et al. [63] and Varshini et al. [64].

### 2.7. Testicular Cholesterol Levels (TCLs)

First, 0.2 mL of testis homogenate was prepared in ferric chloride solution (FeCl_3_ 6H_2_O (0.05 g) in glacial acetic acid), followed by the addition of 3 mL of sulfuric acid, and incubated at room temperature for 20 min [65]. The absorbance was measured at 540 nm on a spectrophotometer against an appropriate blank without testicular homogenate. The measurement of TCLs was expressed as mg/g tissue weight.

### 2.8. Analysis of Indicators of Oxidative Stress

Testis and epididymis (caput, corpus, and cauda) were homogenized in three buffers: (a) The supernatant obtained through homogenization, prepared using phosphate buffer (pH 7.0) under ice-cold conditions, followed by a centrifugation step (10,000× *g* for 30 min at 4 °C), was used for the assay of oxidative stress variables such as lipid peroxidation (LPx; [66], hydrogen peroxide (H_2_O_2_) generation [67], and superoxide anion [68] and antioxidant enzymes like superoxide dismutase (SOD: E.C. 1.15.1.1) [69] and catalase (CAT: E.C. 1.11.1.6) [70], and glutathione-based enzymes like glutathione peroxidase (GPx: E.C. 1.11.1.9) [71] and glutathione reductase (GR: EC 1.6.4.2) [72]; (b) The supernatant obtained through homogenization prepared using ice-cold 1.15% KCl, tris-HCl (10 mM, pH 7.4), followed by a centrifugation step (10,000 rpm for 15 min), was used to determine the protein carbonyl content [73]; and (c) the supernatant obtained via the centrifugation of tissue homogenate in 12% trichloroacetic acid. (1:4 *v*/*v*) was used as the GSH [74] source, wherein the ability to form thiolate anions in a reaction mixture containing 0.25 mM Ellman’s reagent was measured at 412 nm. Detailed protocols for the determination of the selected antioxidant enzymes from testis and epididymal compartments are described in the Supplementary Materials.

### 2.9. Activity of Caspase-3

Testicular caspase-3 activity levels were tested according to the protocol described by Cid et al. [75]. A homogenate of testis prepared in homogenization buffer (HEPES buffer, NaCl 100 mM, DTT 10 mM, EDTA 1 mM, CHAPS 0.1%, glycerol 10%) was incubated at 4 °C for 3 h, followed by a centrifugation step for 10 min at 10,000× *g*. The reaction mixture consisted of fluorogenic substrate 50 µM of 7-amino-4-methylcoumarin (AMC) and 100 µg of protein. The fluorescence intensity was measured using a fluorimeter (Hitachi) at 380 nm (excitation) and 460 nm (emission). The caspase -3 activity measuring units were pmole of AMC liberated per 100 µg protein.

### 2.10. Testicular Steroidogenic Marker Enzymes Assay

The activity levels of 3β- (3β-HSD) (E.C.1.1.1.51) and 17β-Hydroxysteroid dehydrogenase (17β-HSD) (E.C.1.1.1.61) were tested as per the methodology of Bergmeyer [76]. Briefly, the resultant supernatant obtained via centrifugation (10,000× *g* for 1 h) of the testicular (5% W/V) homogenate prepared in 20 mM of ice-cold Tris-HCl buffer (pH 8.2) was used as the enzyme source. Next, 3β-HSD activity was assessed in a reaction mixture of 2.0 mL comprised of sodium pyrophosphate buffer (100 µmoles; pH 9.0), dihydro epiandrosterone, (0.1 µmoles), NAD (0.5 µmoles), and 25 mg of enzyme protein, while 17β-HSD activity was determined in a reaction mixture of 2.0 mL comprised of sodium pyrophosphate buffer (100 µmoles; pH 9.0), steroid substrate androstenedione (0.08 µmoles), NADPH (0.5 µmoles), and 25 mg of enzyme protein. The activity levels of testicular steroidogenic enzymes were measured at 340 nm. The readings were taken over a period of 5 min. at an interval of 20 sec against the reagent blank (without enzyme source). Finally, 3β-HSD was expressed as µmoles of NAD converted to NADH/mg protein/min, and 17β-HSD was expressed as µmoles of NAD converted to NADH/mg protein/min.

### 2.11. Estimation of Proteins

Protein estimation was performed using Lowry’s method [77]. Unknown concentrations of proteins were determined against a standard graph prepared using bovine serum albumin, and suitable blanks were maintained accordingly. The unit in which the amount of protein was expressed was mg/g tissue.

### 2.12. Molecular Studies

RNA quality seems to be critical in transcriptomic assessments. The isolation total RNA from the testis and epididymis, followed by its quality and quantity, were determined spectrophotometrically and by Qubit analysis (Life Technologies, Gaithersburg, MD, USA), respectively. Subsequently, cDNA libraries were obtained via reverse transcription (True seq RNA libraryprep Kit, Illumina, San Diego, CA, USA). An Illumina Hiseq 2000 (Agilent Bioanalyser2100, Carpinteria, CA, USA) in paired end sequencing mode was used as the platform for transcriptomic analyses of qualified libraries to improve the efficiency of the sequences and to accurately match reference genome sequences. Library pool adapted Illumina were prepared by a commercial service (Agrigenome Pvt. Labs Ltd., Hyderabad, Telangana State, India). Only the reads from samples that passed q value ≥ 30 Phred were considered. The pre-processed reads (high quality reads obtained after removing low-quality reads) were compared against the Rat genome (Ensembl database 97; Rnor _6.0.95. gtf.gz) using the default parameters of Hisat2 program (Version 2.0.5). Cufflinks (Version 2.2.1) software with default parameters was used to estimate the expression of the genes; only genes with q-value ≥ 0.05 were considered as differentially expressed. After determining the fold change of genes, a gene enrichment analysis was executed using publicly available standalone tools like DAVID (Version: v6.8) [78], PANTHER (Version: 14.1) [79], and the g: profiler [80] databases.

### 2.13. RT-qPCR Studies to Validate Transcriptomic Data

In this study, we validated nine DEGs, i.e., *17β-HSD, StAR, lhcgr, ar, nfe2l2* or *nrf2*, *casp3,* and *α_1_-Adrenoceptor* with a log_fold value > 1.5 using a randomly selected RT-qPCR. The forward (F: 5′-3′) and reverse (R: 5′-3′) primers used for quantitative real time PCR studies were as follows: *StAR*: F: CGTCGGAGCTCTCTACTTGG; R: CCCAAGGCCTTTTGCATAGC; product size:145 bp), *HSD17β3* (F: CTGGCCTCTGTATAGCCTGTACTCA; R:GTCTTGGTCACCCTGCTGGTAT; product size: 168 bp), *lhcgr* (F: GCATCCGAACCCTTCCAGAT; R: TCGTTATTCATCCCTTGGAAAGC; product size: 124 bp), *ar*(F: GGGGCAATTCGACCATATCTG; R: CCCTTTGGCGTAACCTCCCTT; product size: 278 bp), *nfe2l2* (F: GGTTGCCCACATTCCCAAAC; R: GGCTGGGAATATCCAGGGC; product size: 116 bp), *casp3* (F: TGGACTGCGGTATTGAGACA; R: GCGCAAAGTGACTGGATGAA; product size: 160 bp); α_1_-Adrenoceptor (F: GTAGCCAAGAGAGAAAGCCG and R: CAACCCACCACGATGCCCAG: 212 bp); cholinergic receptor, muscarinic 3; *Chrna3* (F: CCATCTTGCTAGCCTTCATCA and R: TGAAGGACAGAGGTAGAGTAGC; 110 bp) and *GAPDH* (F: GGTCGGAGTGAACGGATT; R: CTCGCTCCTGGAAGATGG; product size: 227 bp).The primers for selected genes were based on previous studies, i.e., *HSD17β, StAR* and *Lhcgr* [81], *ar* [82], *casp3* [83], *Nfe2l2*: [84], and *GAPDH* (house-keeping gene) [85]. The genes that were validated using RT-qPCR were selected for expression studies in PFOA exposed and PFOA plus RES exposed rats.

For qPCR analysis, total RNA was isolated from selected tissues using a Trizol plus purification system, purchased from Invitrogen, Carlsbad, CA, USA, and its purity was determined spectrophotometrically (Model: Jasco v-750; Mary’s Court Easton, MD 2160) and by agarose gel electrophoresis. The quantity of isolated RNA was determined using a NanoDrop-2000 spectrophotometer (Thermo-fisher scientific). The cDNA synthesis of the first strand was performed using an iscript^TM^ cDNA synthesis kit (Biorad, Mumbai, India),m according to the manufacturer’s protocol, and using 1 µg of total RNA. The synthesized cDNA was used to express the mRNA levels of selected genes through RT-qPCR (quantitative real time PCR; Applied Biosystems, Foster City, CA, USA). RT-qPCR assay was carried out using SYRB^TM^ green master mix, 2 µL cDNA, 0.5 µL of each primer (50 nm), and RNase\DNase-free H_2_O up to 20 µL. Prior RT-qPCR analysis, the efficiency of primers was determined and found to be >90%.

The reaction parameters were as follows: first, an initial denaturation step at 95° C for 10 min, followed by 40 cycles at 95 °C for 15 s; then, annealing and extension steps at 60 °C (*Lhcgr*, and *Star*) or 61 °C (*Hsd17b3*) for 30 s and 72 °C for 5 min.; *ar*—50 °C for 15 s and 72 °C for 1 min.; *GAPDH*—51 °C for 15 sec and 72 °C for 1 min.; *casp3*—56 °C for 30 s and 72 °C for 30 s; *nfe2l2*—55 °C for 60 s, extension at 72 °C for 90 s, followed by a final extension step at 72 °C for 10 min. The quantity of DNA was determined based on the standard curves prepared from the cDNA reaction products after serial dilutions. All samples, including a negative control, were run in triplicate, and the mean Ct values were determined accordingly. The relative mRNA expression (Ct values) of selected genes was normalized against *GAPDH* according to the 2^−∆∆Ct^ method [86].

### 2.14. Testicular Histology

A histological analysis of testis was performed according to the method in [87]. After their isolation and after clearing from adhering tissues, selected tissues were fixed individually in Bouin’s solution (picric acid (15 mL): glacial acetic acid (1 mL)) for 24 h, followed by dehydration, clearing, and impregnation in paraffin wax. This was followed by subjecting the fixed specimens to dehydration in an ascending alcoholic series, followed by placing the specimens in paraffin wax. Paraffin wax trimming was performed to obtain 5 µm thickness specimen sections using rotary microtome. Staining of the sectioned specimens was performed using eosin Y and hematoxylin. The histology of stained sections was visualized under a microscope (Hovers microscope; Model no. HV-12TR).

### 2.15. Circulatory Levels of Reproductive Hormones

Serum hormones like testosterone were analyzed via ELISA, i.e., an enzyme linked immunosorbent assay technique (Diametra, Italy), whereas luteinizing hormone (LH) and follicle stimulating hormone (FSH) were determined based on the principle of competitive binding assays (CLIA test Kits). The assays were determined as per the manufacturer’s instructions, without any deviations.

### 2.16. Testosterone

Briefly, serum was added to pre-coated plates with 100 µL testosterone bound to horseradish peroxidase, followed by an incubation step for 1 h at 37 °C. Subsequently, the plates were washed thrice using 300 µL of the wash buffer supplied in the kit. Between every washing step, the plates were shaken gently for 5 s and excess solution was removed. To the plates, 100 µL of substrate (H_2_O_2_-3,3’,5,5’-tetramethylbenzidine) was added and kept in a dark place at room temperature for 15 min., followed by the addition of 100 µL of stop solution consisting of sulfuric acid (0.15 mol/L) to stop the reaction. The absorbance was measured at 450 nm against blanks. All the samples were run at the same time to avoid inter-assay variation. The sensitivity of ELISA was found to be 0–16 ng/mL. Testosterone levels in serum were expressed as ng/mL.

### 2.17. Serum FSH and LH

Briefly, standards or samples were added toCLIA microplates and combined with biotinylated labelled antibodies, followed by the addition of horseradish peroxidase (HRP) conjugate. After the incubation and washing steps, a substrate solution was added to each well. The mixture of gonadotropins, biotin labelled antibodies, and HRP conjugate emitted fluorescence, which was measured by a chemiluminescence immunoassay analyzer as relative light units (RLU). The concentrations of FSH and LH (expressed as ng/mL) in the samples were analyzed from the standard graph prepared from the standards. All the test and standards were run in duplicate, and random tests were repeated with 10% of total samples. The coefficients of variation between the samples and within the samples were less than 10%.

### 2.18. Statistical Analysis

The data in this study are shown as mean ± S.D. The data were analyzed using one way analysis of variance (ANOVA) followed by post-hoc Tukey test (Statistical package for social science version 16.0; SPSS Inc., Chertsey, UK). The results were considered statistically significant at *p* < 0.05. Analyses of transcriptomic data were performed using free, standalone tools like PANTHER and DAVID. The Bonferroni method was adopted for the multiple testing set (*p* < 0.05) and multiple test correction. The Benjamini method and Benjamini-Hochberg method were applied to determine the false discovery rate (FDR) (threshold value: 0.05) in our gene enrichment analysis.

## 3. Results

### 3.1. General Observations

No mortality was observed in the control and experimental groups, and none of the animals was excluded from the study. No signs of clinical toxicity, such as lethargic movements, loss of fur, licking, salivation, or continuous urination were noticed in any of the control or experimental rats. No significant changes were observed in sexual behavior parameters such as number of mounts, latency to the first mount, latency to the first intromission, number of intromissions until first ejaculation, and latency to the first ejaculation between control and experimental groups (Table S1).

### 3.2. Effect of Prepubertal Exposure to PFOA on Fertility in Adult Rats With or Without RES Supplementation

Table 1 presents the fertility efficacy of control and experimental rats. No significant changes were observed in the number of copulation trials, mating index, or fertility index between control and experimental rats. On the other hand, a significant reduction in the average number of implantations/rat (−39.906%; *p* < 0.0001), pre- (43.44% vs. 6.37%) and post- (33.984% vs. 5.94%) implantation loss in females cohabited with PFOA exposed males was observed over controls, while RES supplementation improved the average number of implantations/rat (35.156%; *p* < 0.001), pre- (19.42% vs. 43.44%) and post- (1.637% vs. 33.984%) implantation loss in females mated with PFOA + RES supplemented rats over PFOA alone exposed rats. A significant reduction in the number of live fetuses/rat (−57.820%; *p* < 0.0001) in females cohabited with PFOA treated rats was observed on the 18th day of pregnancy at autopsy as compared to controls. Meanwhile, a significant increase (101.38%; *p* < 0.001) in the number of live fetuses/rat in females cohabited with rats subjected to both PFOA plus RES was observed compared to PFOA alone treated rats. No significant changes were observed in the average number of implantations/rat, pre- and post-implantation loss in females cohabited with control and RES alone treated rats.

### 3.3. Effect of Prepubertal Exposure to PFOA on Body Weight and Tissue Somatic Indices in Adult Rats With or Without RES Supplementation

No significant changes in the body weight or the relative weights of brain, liver, or kidney were observed between control and experimental rats (Table S2). With respect to the relative weights of testis (−50%; *p* < 0.0001) and epididymis (−20.235%; *p* < 0.0001), significant decreases were observed in PFOA exposed rats over controls. RES supplementation showed reversal effects in the relative weights of testis (36.734%, *p* < 0.001) and epididymis (11.21%; *p* < 0.01) in PFOA exposed rats over PFOA alone treated rats (Table S2). No significant changes were noticed in the relative weights of other accessory organs between control and experimental groups (Table S2).

### 3.4. Effect of Prepubertal Exposure to PFOA on Qualitative and Quantitative Sperm Parameters, Testicular Steroidogenesis, and Serum Reproductive Hormones in Adult Rats With or Without RES Supplementation

**Figure 1:**
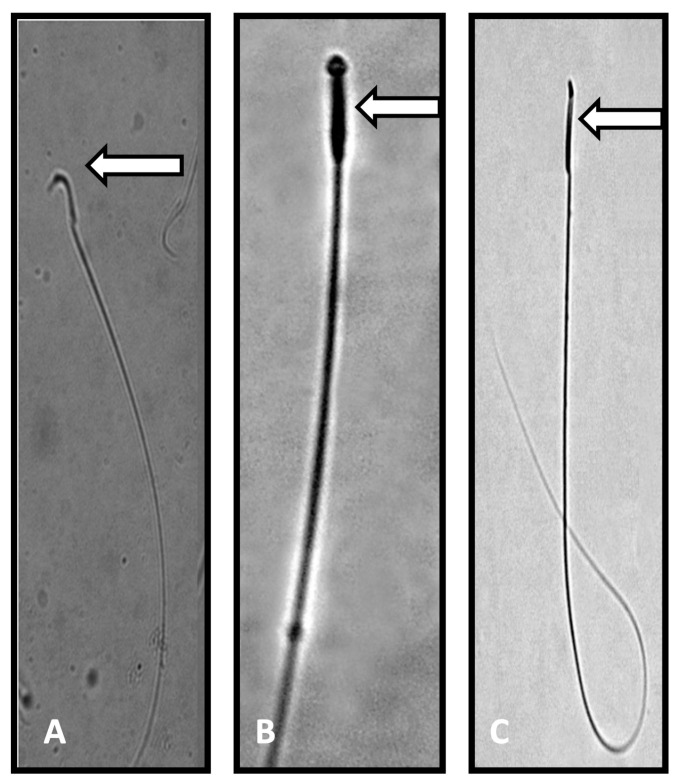
Photomicrograph of sperm from control and Perfluorooctanoic acid (PFOA) exposed rats. (**A**): Normal sperm with a characteristic hook shaped head (control rats). (**B**,**C**): Sperm head abnormalities, such as pin shaped head, no head, or a broken head (PFOA exposed rats). Note: the characteristic hook shape was missing in the sperm of PFOA exposed rats.

**Figure 2:**
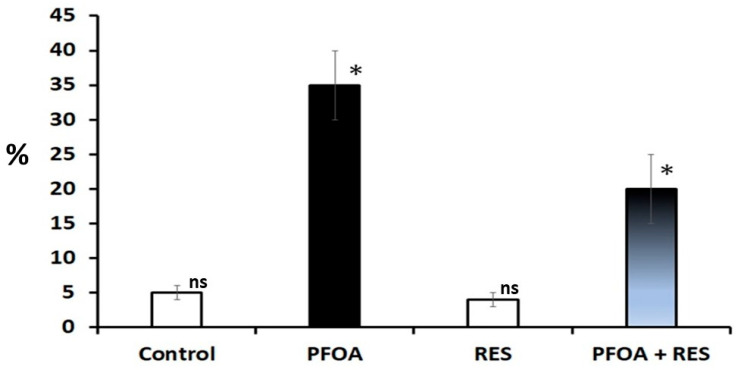
Sperm DNA damage in rats in the control, perfluorooctanoic acid (PFOA) exposed, resveratrol (RES) treated, and PFOA exposed plus RES supplementation groups, as revealed by comet assay. Bars in white did not differ significantly from each other. Asterisks indicate a significant difference at *p* < 0.001 over control. ns = non-significant.

Table 2 summarizes the restorative effects of RES on sperm variables against PFOA induced spermatotoxicity and inhibition of testosterone biosynthesis. A significant reduction in testicular daily sperm count (−51.768%; *p* < 0.0001) and cauda epididymal sperm variables, like sperm count (−46.023%; *p* < 0.0001), motile sperm (−46.879%; *p* < 0.0001), viable sperm (−46.847%; *p* < 0.0001), and tail coiled sperm (−49.407%; *p* < 0.0001) numbers, associated with a significant increase (906.140%; *p* < 0.0001) in the sperm morphological abnormalities (Figure 1), was observed in PFOA exposed rats as compared to controls. RES supplementation showed restoration of testicular daily sperm count (36.277%; *p* < 0.0001) and cauda epididymal sperm count (48.176%; *p* < 0.0001), sperm motility (58.851%; *p* < 0.0001), sperm viability (53.054%; *p* < 0.0001), and number of tail coiled sperm (57.667%; *p* < 0.0001) in PFOA exposed rats over PFOA alone treated rats. RES supplementation showed a reduction (−34.742%; *p* < 0.001) in the sperm head abnormalities in rats exposed to PFOA during the prepubertal period. A significant increase in sperm DNA damage was observed in PFOA exposed rats compared to controls, while RES supplementation ameliorated sperm DNA damage in PFOA exposed rats (−20%; *p* < 0.001) compared to PFOA alone treated rats (Figure 2).

A statistical analysis revealed a significant decrease in the activity levels of 3β-HSD (−52.397%; *p* < 0.0001) and 17β-HSD (−55.307%; *p* < 0.0001) in PFOA exposed rats as compared to controls. Meanwhile, a reversal in the activity levels of testicular steroidogenic enzymes was noticed in RES supplemented PFOA exposed rats (3β-HSD: 68.532%; *p* < 0.01; 17β-HSD: 60.678%; *p* < 0.0001) over PFOA alone treated rats. A similar trend was observed with testicular cholesterol levels (Table 3). A significant decrease in serum testosterone (−52.917%; *p* < 0.0001) levels, accompanied by significant increase in serum FSH (93.107%; *p* < 0.0001), and serum LH (159.715%; *p* < 0.0001) levels, was observed in PFOA exposed rats as compared to controls, suggesting a compromised pituitary-testicular axis. RES supplementation ameliorated the levels of serum testosterone (44.381%; *p* < 0.0001) with a reduction in the circulatory levels of FSH (−30.62%; *p* < 0.0001) and LH (−31.813%; *p* < 0.0001) in RES supplemented PFOA exposed rats compared to PFOA exposed rats, indicating an RES-sustained intact pituitary-testicular axis (Table 3).

### 3.5. Effect of Prepubertal Exposure to PFOA on the Histology of Testis in Adult Rats With or Without RES Supplementation

**Figure 3:**
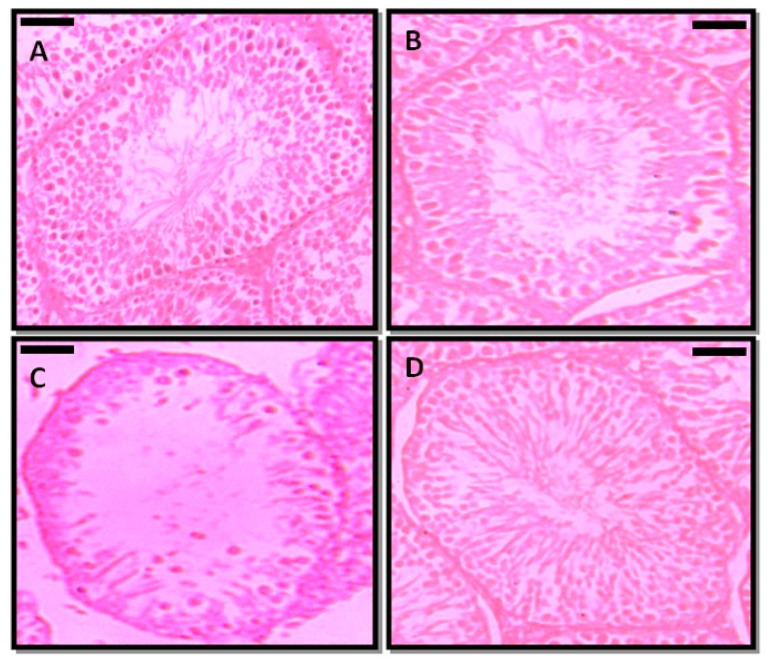
Photomicrographs of the testicular architecture of control (**A**), resveratrol (RES, (**B**)), perfluorooctanoic acid (PFOA, (**C**)), and PFOA plus RES (**D**) treated rats. Scale bar: 50 µm.

The transverse sections of testis were evaluated in PFOA treated and control rats (Figure 3). In controls, the transverse section showed intact seminiferous tubules, and each tubule indicated the various developmental stages of spermatogenic cells. All the tubules were compactly arranged, and each tubule contained Leydig cells and Sertoli cells. The lumens of the tubules were occupied by mature spermatozoa. On the other hand, prepubertal exposure to PFOA showed marked changes in the histology of testis of rats, as indicated by a disrupted basement membrane, i.e., a ruptured epithelial layer, disorganized germ cells, lumen with reduced sperm numbers in the seminiferous tubules, and fewer spermatogenic cells. RES supplementation ameliorated the PFOA induced testicular damage by restoring structural integrity and the lumen occupied with sperm in PFOA treated rats.

### 3.6. Effect of Prepubertal Exposure to PFOA on Pro- and Anti-Oxidant Status in the Testes and Epididymal Regions of Adult Rats With or Without RES Supplementation

Table 4 and Table 5 show pro- and anti-oxidant parameters in the testis, caput epididymis, corpus epididymis, and cauda epididymis, respectively. Prepubertal exposure to PFOA resulted in a significant elevation in LPx (195.642%; *p* < 0.0001), PCs (215.18%; *p* < 0.0001), O_2_^−^ (120.481%; *p* < 0.0001), and H_2_O_2_ (113.776%; *p* < 0.0001) levels in the testis of adult rats compared to controls, while RES supplementation inhibited the levels of oxidative stress parameters (LPx: −36.33%; *p* < 0.001; PCs: −39.86%; *p*<0.001; O_2_^−^: (−33.78%; *p* < 0.001; H_2_O_2_: −28.30%; *p* < 0.001) in the testis of PFOA exposed rats compared to PFOA exposed rats. PFOA intoxication caused region-specific changes in oxidative stress parameters in the epididymis, wherein PCs did not differ significantly in caput and corpus epididymis in adult rats compared to controls, while all regions of the epididymis exhibited significant elevations in LPx, O_2_^−^ and H_2_O_2_ in PFOA exposed rats compared to controls. Enzymatic (SOD: −62.903%; *p* < 0.0001; CAT: −54.166%; *p* < 0.0001; GPx: −57.971%; *p* < 0.0001; GR: −65.371%; *p* < 0.0001) and non-enzymatic (GSH: −58.206%; *p* < 0.0001) testicular antioxidants were down-regulated significantly in PFOA exposed rats compared to controls. PFOA exposure resulted in region-specific changes in selected antioxidants in the epididymis (Caput: SOD (−41.040%; *p* < 0.0001), and CAT (−39.566%; *p* < 0.0001); corpus: (SOD: −29.870%; *p* < 0.0001; CAT: −44.186%; *p* < 0.0001); GPx: (57.142%; *p* < 0.0001); GR: (100.467%; *p* < 0.0001); cauda: SOD: −33.333%; *p* < 0.0001; CAT: −43.455%; *p* < 0.0001; GPx: −54.008%; *p* < 0.0001; GR: −37.595%; *p* < 0.0001) and (GSH: −43.454%; *p* < 0.0001)) in adult rats compared to controls. On the other hand, amelioration of antioxidant parameters due to RES supplementation (testis (SOD: 65.217%, CAT: 77.27%, GPx:48.27%, GR 51.020%, and GSH: 64.15%), caput (SOD: 40.196%, CAT: 61.88%, GPx: −1.65%, GR: −1.208%, and GSH: 0.99%), corpus (SOD: 28.703%, CAT: 54.48%, GPx: −36.11%, GR: −36.596% and GSH: −7.24%) and cauda (SOD: 32.142%, CAT: 52.160%, GPx: 68.34% GR: 52.868%, and GSH: 51.42%)) was observed in PFOA exposed rats compared to PFOA alone treated rats.

### 3.7. Effect of Prepubertal Exposure to Perfluorooctanoic Acid (PFOA) on Activity Levels of Caspase-3 in Rat Testes at Their Adulthood

A significant elevation (51.49%; *p* < 0.0001) in the activity levels of testicular caspase-3 was observed in rats exposed to PFOA over controls (Table 6), while co-treatment of RES in PFOA exposed rats resulted in a reduction (−30.62%; *p* < 0.0001) in the caspase-3 activity levels in the rat testis compared to PFOA alone treated rats.

### 3.8. Gene Enrichment Analysis

A transcriptomic analysis of the testis and epididymis revealed a total of 23,270 and 20,808 genes, respectively, from control rats, out of which 14,992 testicular transcripts and 16,145 epididymal transcripts were mapped using the PANTHER database. Out of 14,992 testicular mapped genes, the number of genes was categorized as follows. Under the categories biological process (BP), molecular function (MF), and cellular component (CC) the number of gene hits were found to be 28,916, 12,477 and 11,917, respectively (Table S3). Meanwhile, out of 16,145 epididymis mapped genes, the number of gene hits related to BP, MF, and CC were found to be 28,167, 11,983, and 11,707, respectively (Table S4).

### 3.9. Differential Gene Expression Analysis

**Figure 4:**
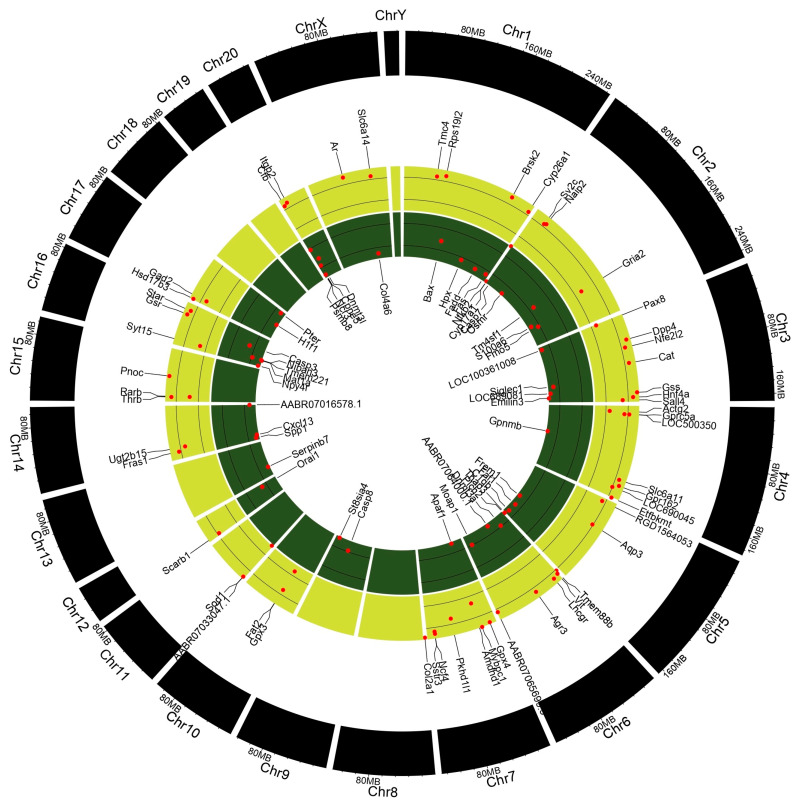
Deregulated genes in the testis of perfluorooctanoic acid exposed rats, shown in the form of Circos plot genes.

A gene expression analysis (GEA) of the testes and epididymis was conducted to identify underlying genetic aberrations following prepubertal exposure to PFOA. Significantly altered genes with a fold change >1.5 relative to control values were subjected to gene enrichment analysis. Testicular transcriptome (Figure 4; Table S5) showed that a total of 98 genes were altered, with 44 up-regulated genes and 54 down-regulated genes, whereas epididymal transcriptome (Table S6) revealed that 611 genes were altered, with 163 up-regulated genes and 448 down-regulated genes in PFOA exposed rats.

**Figure 5:**
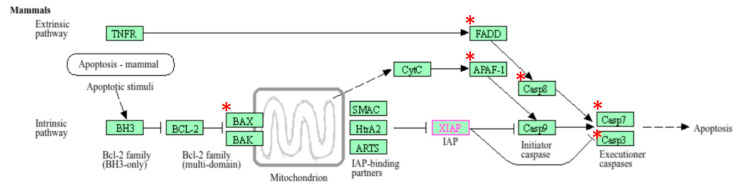
A KEGG pathway analysis of up-regulated genes indicated disruption (top most pathway: fold enrichment value of 98.66) of the apoptosis pathway in the testis of rats exposed to perfluorooctanoic acid during the prepubertal period; * represents genes disrupted in the apoptotic pathway; FADD: Fas associated death domain; APAF1: apoptotic peptidase activating factor 1; BAX: B cell lymphoma 2 associated X; casp7: caspase 7; casp3: caspase 3.

**Figure 6:**
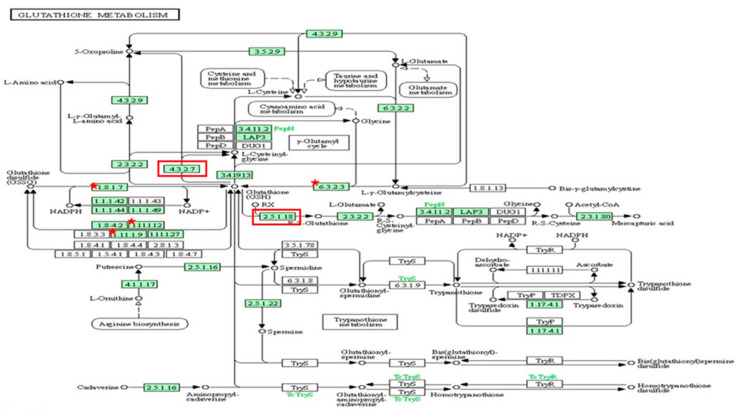
A KEGG pathway analysis of down-regulated genes (Fold enrichment value: 26.8) indicated disruption of the glutathione pathway in the testis and up-regulated genes (Fold enrichment value: 6.4) in the epididymis of rats exposed to perfluorooctanoic acid during the prepubertal period. * represent genes disrupted in glutathione pathway in testis. Glutathione peroxidase 3 (gpx3), glutathione peroxidase 4 (gpx4), glutathione synthetase (gss) and glutathione reductase (gsr). Red colour box represent genes disrupted in glutathione pathway in epididymis. glutathione specific glutamylcyclotransferase 1 (chac1), glutathione S transferase alpha 2 (gst2) and glutathione S transferase alpha 5 (gst5).

Out of 44 up-regulated testicular genes in PFOA exposed rats, 40 genes were mapped through the PANTHER database under different gene ontology terms and sub-categories (13 out of 36 gene hits related to term MF fall under the sub-category catalytic activity (GO: 0003824), 22 out of 85 gene hits related to term BP fall under the sub-category binding (GO: 0005488) and 23 out of 26 gene hits against the term CC fall under the sub-category cellular anatomical entity (GO: 0110165)) (Table S7). A KEGG pathway analysis of testicular up-regulated genes indicated that PFOA exposure led to significant alteration of the apoptosis pathway (Fold enrichment: 98.66; FDR: 3.1 × 10^−0.09^; genes involved: BCL2 associated X, apoptosis regulator (*bax*), Fas associated via death domain (*fadd*), apoptotic peptidase activating factor 1 (*apaf1*), and caspases (*casp3*, *casp7*, *casp8*)) in rats at adulthood (Figure 5; Table 7 and Table S8). On the other hand, out of 54 down-regulated testicular genes in PFOA exposed rats, 48 genes were mapped through the PANTHER database under different gene ontology terms and sub-categories (19 out of 52 gene hits related to term MF fall under the sub-category catalytic activity (GO: 0003824), 26 out of 111 gene hits related to term BP fall under the sub-category cellular process (GO: 0009987) and 23 out of 27 gene hits against the term CC fall under the sub-category cellular anatomical entity (GO: 0110165)) (Table S9). A KEGG pathway analysis of testicular down-regulated genes indicated that PFOA exposure led to a significant alteration in the glutathione pathway (Fold enrichment: 26.8; FDR: 1.4 × 10^−0.03^; Genes involved: glutathione peroxidase 3 (*gpx3*), glutathione peroxidase 4 (*gpx4*), glutathione synthetase (*gss*) and glutathione reductase (*gsr*)) (Figure 6; Table 7 and Table S10) in rats at adulthood.

**Figure 7:**
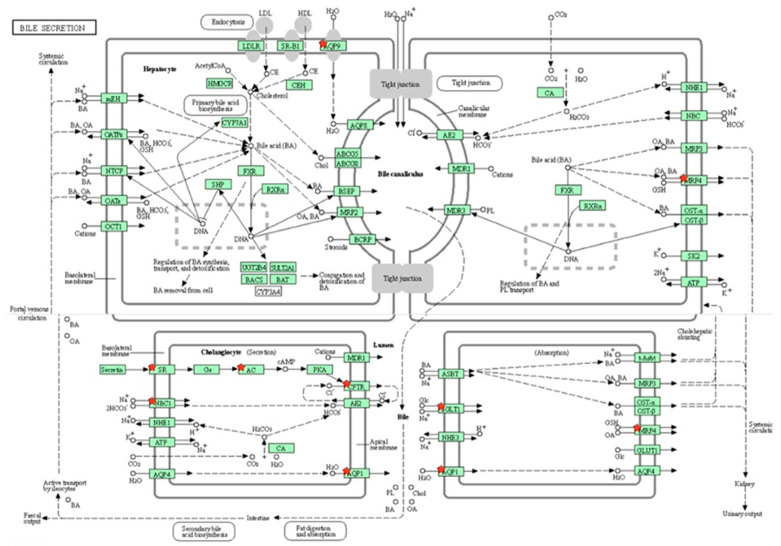
A KEGG pathway analysis of down-regulated genes (Fold enrichment value: 4.4) revealed disruption of the bile secretion pathway in the epididymis of rats exposed to perfluorooctanoic acid during the prepubertal period. * represent genes disrupted in bile secretion pathway. ATP binding cassette subfamily C member 4 (Abcc4), CF transmembrane conductance regulator (Cftr), adenylate cyclase 1(Adcy1), adenylate cyclase 1(Adcy2), aquaporin 1(Aqp1), aquaporin 9 (Aqp 9), secretin receptor (Sctr), solute carrier family 4 member 4(Slc4a4), solute carrier family 5 member 1 (Slc5a1).

Out of 163 up regulated epididymal genes in PFOA exposed rats, 150 genes were mapped through PANTHER database under different gene ontology terms and sub-categories [40 out of 104 gene hits related to term MF fall under the sub-category binding (GO: 0005488), 61 out of 218 gene hits related to term BP fall under the sub-category binding (GO: 0005488) and 77 out of 85 gene hits against the term CC fall under the sub-category cellular anatomical entity (GO: 0110165)] (Table S11). KEGG pathway analysis of testicular up regulated genes indicated that the PFOA exposure led to the significant alteration of glutathione pathway [Fold enrichment: 6.4; FDR: 7.8E-2; Genes involved: glutathione specific glutamylcyclotransferase 1 (*chac1*), glutathione *S* transferase alpha 2 (*gst2*) and glutathione *S* transferase alpha 5 (*gst5*)] in rats at their adulthood (Figure 6; Table 7 and Table S12). On the other hand, out of 448 down regulated epididymal genes in PFOA exposed rats, 448 genes were mapped through PANTHER database under different gene ontology terms and sub-categories [118 out of 373 gene hits related to term MF fall under the sub-category catalytic activity (GO: 0003824), 234 out of 894 gene hits related to term BP fall under the sub-category cellular process (GO: 0009987) and 270 out of 307 gene hits against the term CC fall under the sub-category cellular anatomical entity (GO: 0110165)] (Table S13). KEGG pathway analysis of testicular down regulated genes indicated that the PFOA exposure led to the significant alteration of bile secretion pathway [Fold enrichment: 4.4; FDR: 9.6E-04; Genes involved: *ATP binding cassette subfamily C member 4 (Abcc4), CF transmembrane conductance regulator (Cftr), adenylate cyclase 1(Adcy1), adenylate cyclase 1(Adcy2), aquaporin 1(Aqp1), aquaporin 9 (Aqp 9), secretin receptor (Sctr), solute carrier family 4 member 4(Slc4a4), solute carrier family 5 member 1(Slc5a1)*] (Figure 7; Table 7 and Table S14) in rats at their adulthood.

### 3.10. Validation of RNA-Seq Data Using RT-qPCR

Differentially expressed genes obtained via RNA-Seq analysis were validated by employing RT-qPCR. Nine DEGs were randomly selected that represent testicular and epididymal functions in PFOA exposed rats. A RT-qPCR analysis showed that the expression data obtained via RNA-Seq were correct (Table S15).

### 3.11. Gene Expression Studies

RES supplementation significantly elevated the expression of 17β-HSD (17β-hydroxysteroid dehydrogenase), StAR (Steroidogenic acute regulatory protein), nfe2l2 (nuclear factor erythroid 2 Like 2), ar (androgen receptor), Lhcgr (luteinizing hormone /choriogonadotropin receptor) mRNA and reduced levels of *casp3* (*caspase*-3) mRNA in the testis of PFOA plus RES treated rats compared to PFOA exposed rats. Further, RES supplementation elevated the expression levels of alpha 1 adrenoceptor, muscarinic choline receptor 3, and androgen receptor in the epididymis of PFOA plus RES treated rats compared to PFOA exposed rats (Table 8).

## 4. Discussion

Prepubertal exposure to PFOA at 20 mg/Kg body weight did not show any significant change in the body weight and relative weights of selected non-reproductive tissues, indicating that the general metabolism of the rats was normal. On the other hand, reduced testis and epididymal weights associated with decreased serum testosterone levels in rats exposed to PFOA might reflect that prepubertal period exposure to PFOA may interfere and inhibit androgen synthesis [88]. The observation of elevated levels of cholesterol, associated with reduced activity levels of 3β-and 17β-HSD enzymes in testis of PFOA exposed rats, could be suggestive of improper channeling and conversion of cholesterol to testosterone. These factors could be associated with the inhibition of testosterone production in rats exposed to PFOA [29]. Testosterone is one of the prerequisites for spermatogenesis and its maturation, which occurs in the testis and epididymal regions, respectively. Daily sperm counts are one of the sperm indicators of the spermatogenesis capacity of testis, while the motile nature reflects the degree of maturation of sperm. Therefore, reduced daily sperm counts and post testicular sperm maturation events, accompanied by increased sperm head abnormalities and sperm DNA damage, could be a consequence of inhibition of testicular steroidogenesis in PFOA exposed rats at adulthood [23,24,27,29,89].

Among several factors, it is believed that oxidative stress is one of the causative factors of male infertility [90]. LPx attack lipids through highly reactive forms of ROS like superoxides and hydrogen peroxides formed via Fenton reaction and Haber-Weis Reaction in cellular systems [91], while PCs attack proteins via reactive aldehydes and ketones generated as a result of the oxidation of amino acids by ROS and reactive nitrogen species [92]. In general, the testis and epididymis are endowed with counterattack mechanisms involving enzymatic and non-enzymatic antioxidants to negate the effects of oxidative damage [91,93]. SOD and CAT protect cellular systems against superoxides and hydrogen peroxides, respectively, and are therefore considered to be first defense enzymatic antioxidants. GPx, GR, and reduced GSH act as a glutathione triad system, wherein GPx negate the effects of hydrogen peroxide using glutathione as an electron donor, while GR activity sustains an adequate level of reduced glutathione in cellular systems [94,95]. Our findings suggest that prepubertal exposure to PFOA induces an excess generation of free radicals due to a marked elevation of LPx and PCs, associated with diminished antioxidant levels in the testis and epididymis of rats, leading to oxidative toxicity. Reduced SOD and CAT activities in the testis and cauda epididymis of PFOA exposed rats may, respectively, suggest improper superoxide and hydrogen peroxide removal from cells. A significant reduction in GPx, GR, and GSH levels might reflect a failure of the counterattack mechanism of the glutathione system against peroxides and might be correlated with an inadequate supply of GSH to selected tissues of rats exposed to PFOA. Previously, deteriorated testicular and epididymal functions, associated with elevated oxidative stress, were reported following PFOA intoxication in mice [96] and rats [27]. The authors of [97] showed that male ICR mice exposed to PFOA presented structural damage to the testis and epididymis, a reduction in numbers of spermatogenic cells, elevated levels of 8-hydroxy-2′-desoxyguanosine, and a decrease in GPx and SOD activity levels. The authors of [29] reported that PFOA treatment deteriorated the testicular architecture, associated with elevated levels of LPx and a concomitant decrease in the SOD and GPx in rats. PFOA-induced oxidative damage in liver [27,98,99,100,101], kidney [27], pancreas [102], and brain [103], and even in biological fluids like serum [104], has been reported.

The application of transcriptomic analysis in toxicity studies is commonplace. Developmental exposure to PFOA resulted in a deregulation of genes, associated with the disruption of apoptosis in testis and bile secretion in the epididymis, as well as in glutathione metabolism in both tissue-types in adult rats. Apoptosis in the testis maintains the germ cell rate, the germ cell to Sertoli cell ratio during development, and, ultimately, spermatogenesis later in life [105,106,107]. PFOA exposure caused up-regulation of genes *casp3*, *casp8*, *apaf1*, *fas*, *bax*, and *fadd*, associated with reduced sperm density and androgen synthesis in the testis of rats at adulthood; this may suggest apoptosis of germ cells, Sertoli cells, and Leydig cells. Published reports have indicated that exposure to PFOA causes accelerated apoptosis of germ cells in testis [108] and/or improper maturation of germ cells [3], as evidenced by a loss of different populations of cells, including primary spermatocytes in rat testis. In vitro studies have shown that the possible mechanism underlying PFOA-induced apoptosis could be a triggered expression of pro-apoptotic proteins like p53 and Bax, accompanied by reduced expression of BCl2 and also via Fas mediated death domain [24,109,110,111]. Previously, it has been shown that PFOA is able to induce apoptosis in various tissues like lungs [112], pancreas [113], liver [114], kidneys [18], ovaries [115], placenta [116], and testis [26,117,118] in experimental models. These events eventually lead to the activation of caspase 3, whose activation executes programmed cell death [119]. PFOA exposure resulted in up-regulation of the *caspase 3* gene, which is associated with elevated levels of caspase 3 activity in the testis of rats at adulthood, suggesting the activation of apoptosis.

Transcriptional profiling of the testis (down regulation of *gpx3*, *gpx4*, *gss,* and *gsr*) and epididymis (up regulation of *chac1*, *gst3*, *gst2,* and *gst5)* revealed alterations in genes associated with glutathione metabolism and could be ascribed to developmental toxic effects due to PFOA exposure in adult rats. Glutathione synthetase (encoded by *gss*) is one of the glutathione synthesizing enzymes, while GR is able to catalyze the oxidized form of glutathione to its reduced form, which is eventually utilized by GPx to negate the effects of hydrogen peroxide. In the γ-glutamyl-cycle, γ-glutamyl-cyclotransferases (*chac1*) is essential for maintaining intracellular glutathione levels, while glutathione S-transferases conjugate GSH to electrophiles, thereby reducing their burden in the cellular system. Previously, the toxic effects of PFOA on GSH transferases [120] and GSH peroxidases [121] has been reported. In accordance with our transcriptomic analysis, a significant reduction was observed in the activity levels of the glutathione system in PFOA exposed testis and cauda epididymis of rats. It has been shown that down-regulation of *gpx3* [122] and *gpx4* [123] could be associated with the elevated levels of LPx and ROS in the testis of PFOA treated rats. Even though *nuclear factor erythroid-derived 2-like 2* (*nfe2l2*), a key regulator of expression of antioxidant enzymes such as SOD and CAT [124], is not categorized in the KEGG pathway, RT-qPCR analysis revealed down-regulation of *nfe2l2* in the testis of PFOA exposed adult rats. This might suggest that a PFOA-induced reduction of antioxidant and non-enzymatic antioxidant levels could be entangled with complex molecular networks.

A KEGG pathway analysis revealed that the down-regulated genes, viz., *Abcc4*, *Cftr*, *Adcy1*, *Adcy2, Aqp1*, *Aqp 9*, *Sctr*, *Slc4a4* and *Slc5a1,* were associated with bile secretion in the epididymis of rats exposed to PFOA. It has been shown that mutations in the genes associated with ABC transporters, such as Abcc4 and CFTR, in the epididymis may interfere with male infertility through the malformation of the epididymis [125]. Slc4a4 is one of the electrogenic bicarbonate co-transporters (HCO_3_^-^), which are key for bicarbonate secretion and absorption, as well as sustaining intracellular pH [126]. The fluid milieu of the epididymis exhibits low bicarbonate ion concentrations and low pH, while the microenvironment of the female uterus/oviduct is alkaline, with a high bicarbonate ion concentration [126,127]. Slc4a4 allows spermatozoa to adapt to such high bicarbonate ion concentration environments and supports sperm capacitation events and sperm fertilizing capacity [128]. Aquaporin 9 is key for sperm concentration in the epididymis through the channeling of water in a bidirectional way. Thus, down-regulation of epididymal *abcc4, cftr*, *slc4a4* and *Aqa9* transcripts in PFOA exposed rats could be associated with improper epididymal functions. Published reports have shown that HepGR cells treated with perfluorodecanoic acid exhibited down-regulation of electrogenic bicarbonate transporter Slc4a4, thereby perturbing HCO3^-^ concentrations [129] and PFOA exposure over a period of 28 days, caused down-regulation of aquaporins like 1, 2, and 3, associated with improper renal filtration in the kidney of rats [130].

RES supplementation showed ameliorative effects against reproductive ailments caused of prepubertal exposure to PFOA. This was evidenced by a significant increase in the weights of testis and epididymis, elevated levels of testicular spermatogenesis and steroidogenesis, epididymal sperm maturation events, and reduced testicular damage in RES plus PFOA exposed rats [38,131,132,133]. RES plus PFOA exposed rats showed a reduction in LH and FSH levels, associated with elevated levels of testosterone, suggesting an intact pituitary-testicular axis [41,134,135]. The pro- to anti-oxidant system was also sustained in RES supplemented PFOA rats, as indicated by elevated testicular and region-specific amelioration of antioxidant levels. RES, with its redox characteristics of phenolic OH groups and its electron delocalization, strongly correlate with its antioxidant potential [136]. Thus, RES induced scavenging effects against excess generation of free radicals could negate PFOA mediated oxidative damage in both the tissues of RES plus PFOA exposed rats. Our results are in agreement with those of studies wherein RES supplementation showed recuperative effects on male reproductive health against a range of stressors [42,43,44,45,46,47,137]. The protective effects of RES against perfluorooctanoic sulfonate-induced oxidative damage to the Sertoli cells have therefore been demonstrated [138].

RES induced molecular changes in adult rats exposed to PFOA were also studied using RT-qPCR. Testosterone production and its signaling promote male reproductive tract functions during adulthood. Cholesterol is a precursor of testosterone; two proteins, steroidogenic acute regulatory protein (gene: *StAR*) and scavenger receptor class B member 1 (*scarb1*), channelize cholesterol across the mitochondrial membrane of testis, while 17 β-hydroxysteroid dehydrogenases (*17β-hsd*) catalyzes cholesterol during testosterone biosynthesis [139]. RES treatment showed up-regulation of *star*, *scarb1*, and *17b-HSD* in PFOA exposed rats; this may suggest improved testosterone production via the active channeling of cholesterol and its catabolism events [35]. Androgen receptors are nuclear receptors which mediate the genomic actions of androgens, which are key for spermatogenesis and epididymal sperm maturation events [140]. PFOA induced decreased levels of ar mRNA levels in the testis and epididymis of adult rats, suggesting disturbances at the level of androgen signaling, while RES showed reversal effects on *ar* mRNA levels in PFOA exposed rats. The mRNA levels of antioxidant transcriptional regulator coded by *nfe2l2* was inhibited in the testis of PFOA exposed rats [121,141] but up-regulated in RES plus PFOA exposed rats [142]. The observed elevated levels of the cellular antioxidant enzymes which are under the control of *nfe2l2* in RES supplemented PFOA exposed rats could have been due to the blocking of keap1 proteins by RES [143]. A possible mechanism could be as follows: under normal conditions, nfe2l2 binds with keap1 proteins, followed by ubiquitin degradation, while under oxidative stress conditions, nfe2l2 dissociates with keap1 and promotes downstream activities, such as the activation of cytoprotective genes, including enzymatic antioxidants [144]. Adrenoreceptor alpha 1 and acetylcholine receptors mediate neuroactive signals, i.e., smooth muscle contraction during the ejaculation of spermatozoa from the cauda epididymis [145]. PFOA exposure resulted in decreased mRNA levels of *Alpha adrenoceptor 1* and *Muscarinic acetylcholine receptor 3*, while reversal effects were observed in the cauda epididymis of rats in the RES plus PFOA group. PFOA induced alterations at the level of the purinergic and cholinergic system have been demonstrated in zebrafish [146]. Beneficial effects of RES on learning and memory functions, by acting on muscuranic cholinergic receptors in rats treated with a memory inhibiting drug, i.e., scopolamine, have been reported [147]. We found that an increase in testicular caspase 3 levels in rats exposed to PFOA could be ascribed to RES mediated antiapoptotic effects. RES induced antiapoptotic effects in the epididymis have been demonstrated against several toxic insults, as indicated by the optimization of proapoptotic factor *bax* to anti-apoptotic factor *bcl2* ratio [45,148,149,150,151].

One of our important findings, i.e., that exposure to PFOA deteriorated fertility potential in adult rats exposed during their pre-pubertal period, but that this was ameliorated by co-treatment with RES, might be suggestive of RES induced restoration of spermatogenesis. RES mediated protection of the testis and epididymal regions against PFOA-induced oxidative damage could be a plausible mechanism for improved fertility potential in male rats. Moreover, the polyunsaturated fatty acids (PUFA) present in the plasma membrane of sperm are a vulnerable target of ROS attack [91]. The microenvironment of the epididymal region is also essential to protect sperm against free radical attack. The region specific oxidative damage in the epididymis in PFOA exposed rats may lead to sperm damage and eventually a loss of fertilizing ability [27,29]. RES supplementation restored the testicular and epididymal antioxidant system and brought ROS levels down. This may have protected sperm against oxidative injury and enhanced fertilizing ability in PFOA + RES treated rats. Thus, the observed RES induced improved effects could have been related to (a) antioxidant, (b) antiapoptotic, (c) steroidogenic effects, or (d) all of the above.

Piecing the results together, it can be suggested that the possible mechanism of action of PFOA toxicity occurs on two levels: at the molecular level, through the deregulation of genes associated with apoptosis, antioxidant system, microenvironment of epididymis, which leads to (a) an improper ratio of germ cell to Sertoli cells in testis, (b) Leydig- and Sertoli cell toxicity, (c) deterioration of the antioxidant system and inadequate microenvironment factors in epididymal compartments, or all of the above; or at the biochemical level, through a lack of a counterattack mechanism in the antioxidant system against excess generation of free radicals. One of the interesting questions at this juncture could be how an inert molecule like PFOA triggers oxidative stress. Recent studies by Xu et al. [152] suggested that PFOA is able to directly interact with SOD via hydrophobic bonds, thereby altering its structure and leading to oxidative stress and apoptosis. A similar mechanism may occur with other antioxidant enzymes like CAT, GPx, and GR [153]. These events would result in oxidative damage and apoptosis, as observed in PFOA exposed rats, eventually causing inhibition of testosterone biosynthesis and altered spermiogram parameters, including during post-testicular sperm maturation events. As a consequence of these events, reduced fertility might be observed in rats exposed to PFOA during their prepubertal period. The present rat model system, developed under prepubertal PFOA stress, may act as a system for in-depth analyses at the levels of endocrinology and molecular networks, and could be used at a later date for the development of therapeutic strategies.

There were limitations in this study, especially related to the authentication of the genes obtained via transcriptomic analysis. We only included a limited number of genes for validation. Moreover, we identified transcriptional indicators associated with prepubertal exposure to PFOA induced reproductive toxicity, and further studies are required to authenticate this phenomenon at the protein level. Despite these limitations, our study unequivocally provides relevant findings regarding the developmental toxicity of PFOA in adult rats and the recuperative effects of RES.

## Figures and Tables

**Table 1:** Effect of resveratrol (RES) on in vivo fertility assays in rats exposed to perfluorooctanoic acid (PFOA) during prepubertal period.

Parameter	Control		PFOA Exposed	
	Untreated	RES	Untreated	RES
Number of litters	10	10	10	10
No. of mating trails (days)	1.48 ^a^ ± 0.27	1.52 ^a^ ± 0.31 (2.702)	1.62 ^a^ ± 0.42 (9.459)	1.49 ^a^ ± 0.39 (−1.97); **(−8.02)**
Mating Index (%)	100	100	100	100
Fertility Index (%)	100	100	100	100
No. of corpora lutea/rat	13.65 ^a^ ± 1.43	13.42 ^a^ ± 1.31 (−1.68)	13.58 ^a^ ± 1.48 (−0.51)	13.13 ^a^ ± 1.92 (−3.31); **(−2.160)**
No. of implantations/rat	12.78 ^a^ ± 1.02	12.89 ^a^ ± 1.21 (0.86)	7.68 ^b^ ± 0.42 (−39.906)	10.38 ^c^ ± 0.31 (−19.47); **(35.156)**
Pre implantation loss (%)	6.37	4.11	43.44	19.42
No. of live foetuses/rat	12.02 ^a^ ± 0.39	11.91 ^b^ ± 0.28 (−0.92)	5.07 ^c^ ± 0.18 (−57.820)	10.21 ^d^ ± 0.28 (−14.27); **(101.38)**
Post implantation loss (%)	5.94	7.60	33.984	1.637
Body weight of pups (g) on PND 1	4.81 ^a^ ± 0.53	4.92 ^a^ ± 0.48 (2.286)	4.84 ^a^ ± 0.51 (0.623)	4.89 ^a^ ± 0.49 (−0.609); **(1.03)**
Body weight of pups (g) on PND 21	30.28 ^a^ ± 1.71	29.48 ^a^ ± 1.68 (−2.642)	30.09 ^a^ ± 1.59 (−0.627)	30.07 ^a^ ± 1.74 (2.001); **(−0.066)**

Values are expressed as mean ± S.D. of 10 individual rats. Values in the parentheses are percent changes from control values. Values in the parentheses (bold) are percent changes from those of PFOA exposed rats. Mean values with superscripts with the same letters in a row did not differ significantly from each other at *p* < 0.05.

**Table 2:** Changes in the sperm reproductive endpoints of rats exposed to perfluorooctanoic acid (PFOA) during the prepubertal period, with or without resveratrol (RES) supplementation.

Parameters	Controls		PFOA Exposed	
	untreated	RES	Untreated	RES
Daily sperm count (millions/g epididymis)	26.29 ^a^ ± 1.43	25.76 ^a^ ± 1.62 (−2.015)	12.68 ^b^ ± 2.18 (−51.768)	17.28 ^c^ ± 1.09 (−32.919); **(36.277)**
Sperm analysis (epididymis)				
Sperm count (millions/mL)	74.68 ^a^ ± 4.32	73.91 ^a^ ± 5.21 (−1.031)	40.31 ^b^ ± 4.38 (−46.023)	59.73 ^c^ ± 3.22 (−19.185); **(48.176)**
Sperm viability (%)	73.9 ^a^ ± 3.2	72.98 ^a^ ± 4.33 (−1.244)	39.28 ^b^ ± 4.09 (−46.847)	60.12 ^c^ ± 4.82 (−17.621); **(53.054)**
Sperm motility (%)	70.18 ^a^ ± 4.29	69.33 ^a^ ± 3.47 (−1.211)	37.28 ^b^ ± 3.19 (−46.879)	59.22 ^c^ ± 3.43 (−14.582); **(58.851)**
HOS tail coiled sperm (%)	69.99 ^a^ ± 3.33	68.72 ^a^ ± 2.91 (−1.814)	35.41 ^b^ ± 2.09 (−49.407)	55.83 ^c^ ± 2.19 (−18.757); **(57.667)**
Abnormalities of sperm (%)	2.28 ^a^ ± 0.72	2.92 ^a^ ± 0.16 (28.070)	22.94 ^b^ ± 2.39 (906.140)	14.97 ^c^ ± 3.23 (412.67); **(−34.742)**
Pin shaped (%)	1	1	12	8
Rod shaped (%)	1	1	10	6
Sperm transit time (days)	6.91 ^a^ ± 1.02	7.01 ^a^ ± 1.22 (1.44)	3.84 ^b^ ± 0.83 (−44.42)	5.93 ^c^ ± 0.67 (−14.18); **(35.24)**

Values are expressed as mean ± S.D. of 10 individual rats. Values in parentheses are percent changes from control values. Values in the parentheses (bold) are percent changes from those of PFOA exposed rats. Mean values with superscripts indicating the same letters in a row did not differ significantly from each other at *p* < 0.05.

**Table 3:** Changes in the testicular steroidogenic machinery of rats exposed to perfluorooctanoic acid (PFOA) during the prepubertal period with or without resveratrol (RES) supplementation.

	Controls		PFOA Exposed	
Parameters	Untreated	RES	Untreated	RES
Total cholesterol (mg/g)	6.01 ^a^ ± 0.41	5.92 ^a^ ± 0.31 (−1.497)	10.08 ^b^ ± 0.16 (67.720)	7.32 ^c^ ± 0.16 (23.648); **(−27.3809)**
3β-HSD	16.89 ^a^ ± 2.38	16.46 ^a^ ± 2.24 (−2.545)	8.04 ^b^ ± 1.87 (−52.397)	13.55 ^c^ ± 1.23 (−17.679); **(68.532)**
17βHSD	11.21 ^a^ ± 0.94	11.33 ^a^ ± 0.83 (1.070)	5.01 ^b^ ± 0.32 (−55.307)	8.05 ^c^ ± 0.66 (−28.949); **(60.678)**
T (ng/mL)	7.37 ^a^ ± 0.19	7.22 ^a^ ± 0.23 (−2.035)	3.47 ^b^ ± 0.13 (−52.917)	5.01 ^c^ ± 0.14 (−30.61); **(44.380)**
FSH (ng/mL)	8.27 ^a^ ± 0.91	8.17 ^a^ ± 0.31 (−1.209)	15.97 ^b^ ± 0.81 (93.107)	11.08 ^c^ ± 0.56 (35.62); **(−30.62)**
LH (ng/mL)	6.33 ^a^ ± 0.54	6.62 ^a^ ± 0.67 (4.581)	16.44 ^b^ ± 1.71 (159.715)	11.21 ^c^ ± 1.08 (69.34); **(−31.813)**

Values are expressed as mean ± S.D. of 10 individual rats. Values in the parentheses are percent change from control values. Values in parentheses (bold) are percent changes from those of PFOA exposed rats. Mean values with superscripts with the same letters in a row did not differ significantly from each other at *p* < 0.05.

**Table 4:** Changes in the oxidative stress parameters in the testis and epididymal regions in rats exposed to perfluorooctanoic acid (PFOA) during the prepubertal period, with or without resveratrol (RES) supplementation.

Parameters		Control		PFOA exposed	
		Untreated	RES	Untreated	PFOA + RES
LPx	Testis	9.18 ^a^ ± 2.08	10.11 ^a^ ± 0.98 (10.130)	27.14 ^b^ ± 2.39 (195.642)	17.28 ^c^ ± 1.97 (70.92); **(−36.33)**
	Caput	11.83 ^a^ ± 1.82	10.27 ^a^ ± 1.38 (−13.18)	22.91 ^b^ ± 1.31 (93.660)	14.38 ^c^ ± 0.97 (40.02); **(−37.23)**
	corpus	13.18 ^a^ ± 2.09	12.18 ^a^ ± 1.90 (−7.587)	24.28 ^b^ ± 2.19 (84.218)	16.18 ^c^ ± 0.93 (32.84); **(−33.36)**
	Cauda	10.21 ^a^ ± 1.03	11.27 ^a^ ± 2.03 (10.381)	24.38 ^b^ ± 2.34 (138.785)	15.28 ^c^ ±0.98 (35.58); **(−37.33)**
PC	Testis	1.91 ^a^ ± 0.38	1.78 ^a^ ± 0.21 (−6.806)	6.02 ^c^ ± 0.71 (215.18)	3.62 ^d^ ± 0.29 (103.37); **(−39.86)**
	Caput	1.38 ^a^ ± 0.623	1.53 ^a^ ± 0.531 (10.869)	1.41 ^a^ ± 0.591 (2.173)	1.43 ^a^ ± 0.487 (−6.54); **(1.418)**
	corpus	1.76 ^a^ ± 0.528	1.64 ^a^ ± 0.612 (−6.818)	1.69 ^a^ ± 0.612 (−3.977)	1.68 ^a^ ± 0.712 (2.43); **(−0.59)**
	Cauda	1.49 ^a^ ± 0.209	1.68 ^a^ ± 0.318 (12.751)	3.12 ^b^ ± 0.291 (109.395)	1.51 ^a^ ± 0.337 (−10.12); **(−51.6)**
H_2_ O_2_	Testis	10.38 ^a^ ± 0.42	9.27 ^b^ ± 0.32 (−10.693)	22.19 ^c^ ± 1.38 (113.776)	15.91 ^d^ ± 1.09 (71.63); **(−28.30)**
	Caput	10.13 ^a^ ± 1.28	9.73 ^b^ ± 1.31 (−3.94)	17.33 ^c^ ± 1.228 (71.076)	13.48 ^d^ ± 0.97 (38.54); **(−22.22)**
	corpus	10.13 ^a^ ± 1.28	9.73 ^b^ ± 1.31 (−3.94)	17.33 ^c^ ± 1.228 (71.076)	13.48 ^d^ ± 0.97 (38.54); **(−22.22)**
	Cauda	10.92 ^a^ ± 1.08	11.32 ^a^ ± 1.09 (3.66)	18.39 ^b^ ± 0.981 (68.406)	14.08 ^c^ ± 1.07 (24.38); **(−23.44)**
O^2−^	Testis	4.98 ^a^ ± 0.32	4.72 ^a^ ± 0.19 (−5.22)	10.98 ^c^ ± 0.14 (120.481)	7.27 ^d^ ± 0.18 (54.025); **(−33.78)**
	Caput	5.17 ^a^ ± 0.431	4.89 ^a^ ± 0.581 (−5.415)	7.82 ^b^ ± 0.281 (51.257)	5.23 ^c^ ± 0.61 (6.95); **(−33.12)**
	corpus	4.27 ^a^ ± 0.312	4.31 ^a^ ± 0.302 (0.936)	8.31 ^b^ ± 0.273 (94.613)	5.18 ^c^ ± 0.428 (20.18); **(−37.66)**
	Cauda	4.39 ^a^ ± 0.414	4.41 ^a^ ± 0.281 (0.455)	8.21 ^b^ ± 0.314 (87.015)	5.21 ^c^ ± 0.513 (18.14); **(−36.54)**

Values are expressed as mean ± S.D. of 10 individual rats. Values in the parentheses are percent changes from control values. Values in parentheses (bold) are percent changes from those of PFOA exposed rats. Mean values with superscripts indicating the same letters in a row did not differ significantly from each other at *p* < 0.05. Units: lipid peroxidation (LPx): (μmol of malondialdehyde formed/g tissue); hydrogen peroxide generation assay (H_2_O_2_): nmol /mg protein/min; superoxide anion (O^2−^): nmol/mg protein/min; protein carbonyl (PC): nmol carbonyl/mg protein.

**Table 5:** Changes in the antioxidant levels in the testis and epididymal regions of rats exposed to perfluorooctanoic acid (PFOA) during prepubertal period with or without resveratrol (RES) supplementation.

Parameters		Controls		Treated	
		Control	RES	PFOA	PFOA +RES
SOD	Testis	0.62 ^a^ ± 0.15	0.58 ^a^ ± 0.11 (−6.45)	0.23 ^c^ ± 0.12 (−62.903)	0.38 ^d^ ± 0.18 (−34.48); **(65.217)**
	Caput	1.73 ^a^ ± 0.072	1.68 ^a^ ± 0.061 (−2.89)	1.02 ^b^ ± 0.023 (−41.040)	1.43 ^c^ ± 0.029 (−14.88); **(40.196)**
	corpus	1.54 ^a^ ± 0.033	1.52 ^a^ ± 0.042 (−1.29)	1.08 ^c^ ± 0.019 (−29.870)	1.39 ^d^ ± 0.013 (−8.552); **(28.703)**
	Cauda	1.68 ^a^ ± 0.041	1.71 ^a^ ± 0.038 (1.785)	1.12 ^b^ ± 0.017 (−33.333)	1.48 ^c^ ± 0.019 (−13.45); **(32.142)**
CAT	Testis	0.48 ^a^ ± 0.013	0.51 ^a^ ± 0.041 (6.25)	0.22 ^c^ ± 0.014 (−54.166)	0.39 ^d^ ± 0.009 (−23.52); **(77.27)**
	Caput	5.08 ^a^ ± 0.47	5.18 ^a^ ± 0.37 (1.968)	3.07 ^b^ ± 0.21 (−39.566)	4.97 ^a^ ± 0.38 (−4.05); **(61.88)**
	corpus	5.59 ^a^ ± 0.38	6.02 ^a^ ± 0.44 (7.692)	3.12 ^b^ ± 0.29 (−44.186)	4.82 ^c^ ± 0.37 (−19.93); **(54.48)**
	Cauda	5.73 ^a^ ± 0.39	6.12 ^a^ ± 0.51 (6.806)	3.24 ^b^ ± 0.23 (−43.455)	4.93 ^c^ ± 0.30 (−19.44); **(52.160)**
GR	Testis	2.83 ^a^ ± 0.014	2.81 ^a^ ± 0.013 (−0.71)	0.98 ^c^ ± 0.021 (−65.371)	1.48 ^d^ ± 0.015 (−47.33); **(51.020)**
	Caput	3.28 ^a^ ± 0.031	3.25 ^a^ ± 0.040 (−0.91)	3.31 ^a^ ± 0.039 (0.914)	3.27 ^a^ ± 0.049 (0.615); **(−1.208)**
	corpus	2.14 ^a^ ± 0.012	2.19 ^a^ ± 0.022 (2.23)	4.29 ^c^ ± 0.027 (100.467)	2.72 ^d^ ± 0.039 (24.200); **(−36.596)**
	Cauda	3.91 ^a^ ± 0.017	3.95 ^a^ ± 0.043 (1.02)	2.44 ^c^ ± 0.031 (−37.595)	3.73 ^d^ ± 0.024 (−5.569); **(52.868)**
GPx	Testis	2.07 ^a^ ± 0.014	2.04 ^a^ ± 0.012 (−1.44)	0.87 ^c^ ± 0.011 (−57.971)	1.29 ^d^ ± 0.009 (−36.76); **(48.27)**
	Caput	6.02 ^a^ ± 0.042	5.98 ^a^ ± 0.053 (−0.66)	6.05 ^a^ ± 0.067 (0.49)	5.95 ^a^ ± 0.081 (−0.501); **(−1.65)**
	corpus	5.11 ^a^ ± 0.039	5.13 ^a^ ± 0.023 (0.391)	8.03 ^c^ ± 0.033 (57.142)	5.13 ^a^ ± 0.063 (0); **(−36.11)**
	Cauda	4.74 ^a^ ± 0.051	4.79 ^a^ ± 0.024 (1.054)	2.18 ^c^ ± 0.019 (−54.008)	3.67 ^d^ ± 0.020 (−23.38); **(68.34)**
GSH	Testis	10.48 ^a^ ± 1.21	11.29 ^a^ ± 2.08 (7.729)	4.38 ^b^ ± 0.82 (−58.206)	7.19 ^c^ ± 1.07 (−36.32); **(64.15)**
	Caput	9.38 ^a^ ± 0.29	9.48 ^a^ ± 0.33 (1.066)	10.02 ^b^ ± 0.42 (6.823)	10.12 ^c^ ± 0.36 (6.75); **(0.99)**
	corpus	10.23 ^a^ ± 0.41	10.71 ^a^ ± 0.39 (4.692)	11.18 ^b^ ± 0.46 (9.286)	10.37 ^a^ ± 0.32 (−3.17); **(−7.24)**
	Cauda	9.32 ^a^ ± 0.23	9.30 ^a^ ± 0.29 (−0.22)	5.27 ^c^ ± 0.17 (−43.454)	7.98 ^d^ ± 0.20 (−14.19); **(51.42)**

Values are expressed as mean ± S.D. of 10 individual rats. Values in parentheses are percent changes from control values. Values in parentheses (bold) are percent changes from those of PFOA exposed rats. Mean values with superscripts indicating the same letters in a row did not differ significantly from each other at *p* < 0.05. Superoxide dismutase: (SOD: Units/mg protein); catalase (CAT: μmol of H_2_O_2_ decomposed/mg protein/min); glutathione reductase (GR: μmol of NADPH oxidized/mg protein/min); glutathione peroxidase (GPx: μmol of NADPH oxidized/mg protein/min); reduced glutathione (GSH: μmol of thiourea/mg protein/hr).

**Table 6:** Effect of prepubertal exposure to perfluorooctanoic acid (PFOA) on activity levels of caspase 3 in rat testis at adulthood.

Controls		PFOA exposed	
Untreated	RES	Untreated	RES
190.42 ^a^ ± 20.74	192.78 ^a^ ± 30.44 (1.239)	392.48 ^b^ ± 29.48 (106.112)	272.28 ^c^ ± 22.53 (41.23); **(−30.62)**

Values are expressed as mean ± S.D. of 10 individual rats. Values in parentheses are percent changes from control values. Values in parentheses (bold) are percent changes from those of PFOA exposed rats. Mean values with superscripts indicating the same letters in a row did not differ significantly from each other at *p* < 0.05.

**Table 7:** A KEGG Pathway analysis revealed disrupted pathways in the testis and epididymis of adult rats exposed to perfluorooctanoic acid (PFOA) during their prepubertal period.

Term	Count	%	*p*-Value	Fold Enrichment
Testis				
rno04215: Apoptosis—multiple species (up-regulated)	6	14.28571	1.65 × 10^−08^	67.46324
rno00480: Glutathione metabolism (down-regulated)−pididymis	4	8.695652	0.001779	15.92882
rno00480: Glutathione metabolism (up-regulated)	3	1.923077	0.048064	6.371528
rno04976: Bile secretion (down-regulated)	9	2.122642	9.61 × 10^−04^	4.392287

**Table 8:** Changes in the expression of selected genes in the testis and epididymis of rats exposed to perfluorooctanoic acid (PFOA) during the prepubertal period, with or without resveratrol (RES) supplementation.

	Fold Change *	Fold Change *#
Gene Symbol	PFOA	PFOA + RES
***Testis***		
***hsd17β3***	−2.52 ± 0.04	1.23 ± 0.09 (−148.81)
***StAR***	−1.84 ± 0.07	1.45 ± 0.11 (−178.80)
***AR***	−2.04 ± 0.08	0.94 ± 0.14 (−146.07)
***Casp3***	2.67 ± 0.09	1.02 ± 0.24 (−61.79)
***Nfe2l2***	−2.07 ± 0.03	1.55 ± 0.13 (−174.87)
***lhcgr***	−2.79 ± 0.07	1.21 ± 0.11 (−143.36)
***Epididymis***		
***AR***	−1.97 ± 0.05	0.96 ± 0.13 (−148.73)
***Alpha adrenoceptor 1***	−3.18 ± 0.06	0.87 ± 0.13 (−127.35)
***Muscarinic acetylcholine receptor 3***	−2.64 ± 0.11	1.11 ± 0.15 (−142.04)

Fold change refers to gene expression in the testis and epididymis of PFOA exposed and PFOA plus RES treated rats as compared to untreated controls using qPCR (sample size: 05). Values in parentheses are percent changes from those of PFOA exposed rats. For percent change calculations, for PFOA + RES treated rats, PFOA exposed rats served as controls; * Represents a statistically significant difference at *p* < 0.001. For evaluations of statistical significance ‘*p*’ for PFOA exposed rats and PFOA + RES treated rats, untreated rats served as controls. # Represents a statistically significant difference at *p* < 0.001. For evaluations of statistical significance ‘*p*’ for PFOA + RES treated rats, PFOA exposed rats served as controls.

## Data Availability

The data was shared as Supplementary Materials. Three standalone public databases were used and mentioned in Materials and Methods section. The ethical statement was shown in institutional Review Board Statement.

## Supplementary Materials

The following supporting information can be downloaded at: https://www.mdpi.com/article/10.3390/toxics13020111/s1, Table S1: Effect of resveratrol (RES) on selected sexual behaviour parameters in rats exposed to perfluorooctanoic acid (PFOA) during prepubertal period.; Table S2: Changes in the tissue somatic indices (W/W%) of rats exposed to perfluorooctanoic acid (PFOA) during prepubertal period supplemented with or without resveratrol (RES) .; Table S3: Summary of gene ontology of testicular transcriptome from control rats.; Table S4: Summary of gene ontology of epididymis transcriptome from control rat.; Table S5: Summary of deregulated genes in the testis of rats exposed to perfluorooctanoic acid during prepubertal period.; Table S6: Summary of deregulated genes in the epididymis of rats exposed to perfluorooctanoic acid during prepubertal period.; Table S7: Summary of gene ontology of up regulated genes in the testis of rats exposed to perfluorooctanoic acid.; Table S8: KEGG pathway analysis of up regulated genes in the testis of rats exposed to perfluorooctanoic acid during prepubertal period.; Table S9: Summary of gene ontology of down regulated genes in the testis of rats exposed to perfluorooctanoic acid.; Table S10: KEGG pathway analysis of down regulated genes in the testis of rats exposed to perfluorooctanoic acid during prepubertal period.; Table S11: Summary of gene ontology of up regulated genes in the epididymis of rats exposed to perfluorooctanoic acid.; Table S12: KEGG pathway analysis of up regulated genes in the epididiymis of rats exposed to perfluorooctanoic acid during prepubertal period.; Table S13: Summary of gene ontology of down regulated in the epididymis of rats exposed to perfluorooctanoic acid during prepubertal period.; Table S14: KEGG pathway analysis of down regulated genes in the epididiymis of rats exposed to perfluorooctanoic acid during prepubertal period.; Table S15: Differentially expressed genes in the testis and epididymis of rats exposed to perfluorooctanoic acid during prepubertal period.
