# Supplementary material for: Protective Effects of Resveratrol Against Perfluorooctanoic Acid-Induced Testicular and Epididymal Toxicity in Adult Rats Exposed During Their Prepubertal Period
Source: Toxics. 2025 Jan 29;13(2):111. doi: 10.3390/toxics13020111 (PMC11860439; doi:10.3390/toxics13020111)
Supplement: Supplementary file 1 [file toxics-13-00111-s001.zip › toxics-3366247-supplementary.pdf]

Supplementary material

1. Fertility assay parameters were performed as per the following formulas:

   Mating index: [(Number of sperm positive females/Number of paired rats) X 100]

   fertility index: [(Number of pregnant females/Number of sperm positive females) X 100] Pre-implantation loss: [(No. of corpora lutea – No. of implantations/No. of corpora lutea) X 100)

   Post-implantation loss: [(No. of implantations– No. of live foetuses/No. of implantations) X 100] implantation loss

2. Detailed protocol for the determination of lipid peroxidation, superoxide dismutase, catalase, glutathione peroxidase, glutathione reductase from the testis and epididymis of rats under different experimental conditions:

Lipid peroxidation (LPx) assay:

LPx was determined through the formation of concentration of malondialdehyde (MDA), a reactive product of thiobarbituric acid which was measured spectrophotometrically at 535 nm. The rate of lipid peroxidation was expressed as µmoles of malondialdehyde formed/g wet wt. of tissue using extinction coefficient of MDA $1.56 \times 10^5 M^{-1} cm^{-1}$. The generation of H_2O_2 was determined using a reaction mixture of 50 mM phosphate buffer (pH 7.6), 8.5 UI/ml horse radish peroxidase, 0.28 nM phenol red, 5.5 nM dextrose and 600 µl of tissue homogenate. The absorbance was measured at 610 nm on a spectrophotometer. The generation of hydrogen peroxide levels was expressed as nmol per mg protein per min. Superoxide anion production by testis and epididymal tissues was based on the reduction of iodonitrotetrazolium violet and measured spectrophotometrically at 505 nm. The reaction mixture of 1 ml contained 25 µl of the enzyme source, 4.9 mM INT, 0.3 mM ethylene diamine tetra acetic acid (EDTA)

and 0.92 mM sodium carbonate (Na_2CO_3). The amount of superoxide was expressed as nmol per mg protein per min.

Superoxide dismutase (SOD) activity

SOD activity was determined based on the principle that the ability of SOD to inhibit the autoxidation of epinephrine at an alkaline medium which was monitored spectrophotometrically at 480 nm (Analytical Technologies; Model No: 2060) against a suitable blank. The enzyme assays were performed in accordance to the zero-order kinetics following preliminary standardization regarding linearity with respect to enzyme concentration and substrate used. The SOD activity was calculated as Units/mg protein. CAT activity was determined based on the principle that the ability of CAT to decompose hydrogen peroxide concentration at 240 nm. The CAT activity was represented as micromoles H_2O_2 decomposed/mg protein/min.

Glutathione peroxidase (GPx) and glutathione reductase (GR) activity:

GPx and GR activities were determined based on the dismutation of *tert*-butylhydroperoxide at 340 nm via glutathione/ NADPH glutathione reductase system, while GR activity was analyzed through oxidation rate of NADPH in a reaction medium containing oxidized glutathione in a buffer. The activity of GPx expressed as µM NADPH oxidized per mg protein per minute, and the activity of GR was expressed as µmoles of NADPH per mg protein per min.

Table S1: Effect of resveratrol (RES) on selected sexual behaviour parameters in rats exposed to perfluorooctanoic acid (PFOA) during prepubertal period.

**Parameter**	**Controls**		**PFOA exposed**	
	Untreated	RES	Untreated	RES
Latency to the first mount (s)	60.72[a] ± 10.67	62.18[a] ± 11.21 (2.404)	62.38[a] ± 12.18 (2.733)	60.33[a] ± 10.91 (-2.975); **(-3.286)**
Latency to the first intromission (s)	83.22[a] ± 6.72	81.68[a] ± 7.82 (-1.850)	80.97[a] ± 9.68 (-2.703)	83.48[a] ± 7.99 (2.203); **(3.099)**
No. of intromissions until first ejaculation (days)	30.28[a] ± 6.45	29.99[a] ± 7.28 (-0.957)	31.67[a] ± 8.12 (4.590)	30.07[a] ± 7.18 (0.266); **(-5.052)**
Latency to the first ejaculation (s)	650.73[a] ± 60.82	652.81[a] ± 70.91 (0.319)	692.38[a] ± 80.60 (6.400)	701.71[a] ± 90.18 (7.490); **(1.347)**

Values are expressed as mean ± S.D. of 10 individual rats
Values in the parentheses are percent change from that of control. Whereas, values in the parentheses (bold) are percent change from that of PFOA exposed rats.
Mean values with same alphabets in a row did not differ significantly from each other at $p < 0.05$.

Table S2: Changes in the tissue somatic indices (W/W%) of rats exposed to perfluorooctanoic acid (PFOA) during prepubertal period supplemented with or without resveratrol (RES)

Tissue	Control		PFOA exposed	
	Untreated	RES	Untreated	RES
Testes	0.98[a] ± 0.086	1.007[a] ± 0.078 (2.755)	0.49[b] ± 0.06 (-50)	0.67[c] ± 0.056 (-33.465); **(36.73)**
Epididymis	1.018[a] ± 0.018	1.002[a] ± 0.012 (-1.571)	0.812[b] ± 0.011 (-20.23)	0.903[c] ± 0.016 (-9.880); **(11.206)**
Vas deferens	0.153[a] ± 0.021	0.156[a] ± 0.018 (1.960)	0.158[a] ± 0.019 (3.267)	0.155[a] ± 0.020 (-0.641); **(-1.898)**
Prostate gland	0.149[a] ± 0.023	0.151[a] ± 0.017 (1.342)	0.148[a] ± 0.021 (-0.671)	0.150[a] ± 0.019 (-0.662); **(1.351)**
Seminal vesicle	0.452[a] ± 0.031	0.461[b] ± 0.028 (1.991)	0.458[a] ± 0.021 (1.327)	0.449[a] ± 0.035 (-2.603); **(-1.965)**
Brain	0.861[a] ± 0.09	0.875[a] ± 0.100 (1.626)	0.809[a] ± 0.14 (-6.039)	0.820[a] ± 0.100 (-6.285); **(1.359)**
Liver	3.198[a] ± 0.135	3.115[a] ± 0.155 (-2.595)	2.903[a] ± 0.320 (-9.224)	2.919[a] ± 0.275 (-6.292); **(0.551)**
Kidney	0.807[a] ± 0.090	0.806[a] ± 0.180 (-0.123)	0.772[a] ± 0.091 (-4.337)	0.763[a] ± 0.089 (-5.334); **(-1.165)**

Values are expressed as mean ± S.D. of 10 individual rats

Values in the parentheses are percent change from that of control. Whereas, values in the parentheses (bold) are percent change from that of PFOA exposed rats.

Mean values with same alphabets in a row did not differ significantly from each other at $p < 0.05$.

Table 3: Summary of gene ontology of testicular transcriptome from control rats

			**%#**
**S.No**	**Gene ontology term: Biological process**	**No. of genes**	3.80%
1	developmental process (GO:0032502)	1106	4.10%
2	multicellular organismal process (GO:0032501)	1194	29.80%
3	cellular process (GO:0009987)	8605	0.70%
4	reproduction (GO:0000003)	189	7.40%
5	localization (GO:0051179)	2134	0.70%
6	reproductive process (GO:0022414)	189	1.10%
7	biological adhesion (GO:0022610)	314	1.60%
8	immune system process (GO:0002376)	462	15.80%
9	biological regulation (GO:0065007)	4577	0.20%
10	growth (GO:0040007)	71	6.50%
11	signaling (GO:0023052)	1887	17.60%
12	metabolic process (GO:0008152)	5076	0.60%
13	biological process involved in interspecies interaction between organisms (GO:0044419)	187	8.60%
14	response to stimulus (GO:0050896)	2489	0.00%
15	pigmentation (GO:0043473)	12	0.00%
16	biomineralization (GO:0110148)	7	0.20%
17	biological phase (GO:0044848)	45	0.00%
18	behavior (GO:0007610)	9	0.10%
19	rhythmic process (GO:0048511)	23	1.20%
20	locomotion (GO:0040011)	340	
			**%#**
**S.No**	**Gene ontology term: Molecular Function**	**No. of genes**	5.60%
1	transporter activity (GO:0005215)	700	0.50%
2	translation regulator activity (GO:0045182)	65	7.50%
3	transcription regulator activity (GO:0140110)	939	28.20%
4	catalytic activity (GO:0003824)	3523	0.50%
5	cytoskeletal motor activity (GO:0003774)	60	5.30%
6	molecular function regulator (GO:0098772)	658	2.10%
7	ATP-dependent activity (GO:0140657)	266	0.00%
8	low-density lipoprotein particle receptor activity (GO:0005041)	4	7.30%
9	molecular transducer activity (GO:0060089)	909	0.90%
10	molecular adaptor activity (GO:0060090)	114	2.20%
11	structural molecule activity (GO:0005198)	277	39.80%
12	binding (GO:0005488)	4962	
			**%#**
**S.No**	**Gene ontology term: Cellular component**	**No. of genes**	77.70%
1	cellular anatomical entity (GO:0110165)	9254	22.30%
2	protein-containing complex (GO:0032991)	2663	**%#**

# indicates percent calculation against total number of genes under gene ontology term

Table S4: Summary of gene ontology of epididymis transcriptome from control rat.

S.No	Gene ontology term: Biological process	No. of genes	%#
1	developmental process (GO:0032502)	1063	3.80%
2	multicellular organismal process (GO:0032501)	1104	3.90%
3	cellular process (GO:0009987)	8394	29.80%
4	reproduction (GO:0000003)	168	0.60%
5	localization (GO:0051179)	2099	7.50%
6	reproductive process (GO:0022414)	168	0.60%
7	biological adhesion (GO:0022610)	308	1.10%
8	immune system process (GO:0002376)	490	1.70%
9	biological regulation (GO:0065007)	4442	15.80%
10	growth (GO:0040007)	73	0.30%
11	signaling (GO:0023052)	1821	6.50%
12	metabolic process (GO:0008152)	4946	17.60%
13	biological process involved in interspecies interaction between organisms (GO:0044419)	217	0.80%
14	response to stimulus (GO:0050896)	2432	8.60%
15	pigmentation (GO:0043473)	11	0.00%
16	biomineralization (GO:0110148)	9	0.00%
17	biological phase (GO:0044848)	43	0.20%
18	behavior (GO:0007610)	11	0.00%
19	rhythmic process (GO:0048511)	20	0.10%
20	locomotion (GO:0040011)	348	1.20%
**S.No**	**Gene ontology term: Molecular Function**	**No. of genes**	**%#**
1	transporter activity (GO:0005215)	661	5.50%
2	translation regulator activity (GO:0045182)	67	0.60%
3	transcription regulator activity (GO:0140110)	895	7.50%
4	catalytic activity (GO:0003824)	3480	29.00%
5	cytoskeletal motor activity (GO:0003774)	59	0.50%
6	molecular function regulator (GO:0098772)	646	5.40%
7	ATP-dependent activity (GO:0140657)	254	2.10%
8	low-density lipoprotein particle receptor activity (GO:0005041)	4	0.00%
9	molecular transducer activity (GO:0060089)	766	6.40%
10	molecular adaptor activity (GO:0060090)	115	1.00%
11	structural molecule activity (GO:0005198)	257	2.10%
12	binding (GO:0005488)	4779	39.90%
**S.No**	**Gene ontology term: Cellular component**	**No. of genes**	**%#**
1	cellular anatomical entity (GO:0110165)	9104	77.80%
2	protein-containing complex (GO:0032991)	2603	22.20%

# indicates percent calculation against total number of genes under gene ontology term, respectively.

Table S5: Summary of deregulated genes in the testis of rats exposed to perfluorooctanoic acid during prepubertal period

Gene	Ensembl ID	Entrez ID	Chr	Name of the gene
**Up regulated genes**				
LOC100361008	ENSRNOG00000022490	1.00E+08	3	Cytochrome c oxidase subunit 5A, mitochondrial-like
Bax	ENSRNOG00000020876	24887	1	BCL2 associated X, apoptosis regulator
Hpx	ENSRNOG00000018257	58917	1	hemopexin
Fadd	ENSRNOG00000047035	266610	1	Fas associated via death domain
Fas	ENSRNOG00000019142	246097	1	Fas cell surface death receptor
Nfkb2	ENSRNOG00000019311	309452	1	nuclear factor kappa B subunit 2
Cyp17a1	ENSRNOG00000020035	25146	1	cytochrome P450, family 17, subfamily a, polypeptide 1
Casp7	ENSRNOG00000056216	64026	1	caspase 7
Osmr	ENSRNOG00000033192	310132	2	oncostatin M receptor
Tm4sf1	ENSRNOG00000015812	295061	2	transmembrane 4 L six family member 1
S100a6	ENSRNOG00000011647	85247	2	S100 calcium binding protein A6
Fmo5	ENSRNOG00000018076	246248	2	flavin containing dimethylaniline monoxygenase 5
Siglec1	ENSRNOG00000021243	311426	3	sialic acid binding Ig like lectin 1
LOC689081	ENSRNOG00000005178	689081	3	similar to cystatin E2
Emilin3	ENSRNOG00000016734	362262	3	elastin microfibril interfacer 3
Gpnmb	ENSRNOG00000008816	113955	4	glycoprotein nmb
Frem1	ENSRNOG00000022309	298185	5	Fras1 related extracellular matrix 1
Faf1	ENSRNOG00000008523	140657	5	Fas associated factor 1
C1qb	ENSRNOG00000012749	29687	5	complement C1q B chain
Thap3	ENSRNOG00000026840	362667	5	THAP domain containing 3
Plch2	ENSRNOG00000014226	313756	5	phospholipase C, eta 2
Dnmt3a	ENSRNOG00000026649	444984	6	DNA methyltransferase 3 alpha
Moap1	ENSRNOG00000033970	299261	6	modulator of apoptosis 1
Apaf1	ENSRNOG00000008022	78963	7	apoptotic peptidase activating factor 1
Casp8	ENSRNOG00000012331	64044	9	caspase 8
St8sia4	ENSRNOG00000019128	116696	9	ST8 alpha-N-acetyl-neuraminide alpha-2,8-sialyltransferase 4
Orai1	ENSRNOG00000001336	304496	12	ORAI calcium release-activated calcium modulator 1
Serpinb7	ENSRNOG00000002555	117092	13	serpin family B member 7
Spp1	ENSRNOG00000043451	25353	14	secreted phosphoprotein 1
Cxcl13	ENSRNOG00000024899	498335	14	C-X-C motif chemokine ligand 13
Npy4r	ENSRNOG00000061026	29471	16	neuropeptide Y receptor Y4
Mat1a	ENSRNOG00000011351	25331	16	methionine adenosyltransferase 1A
Tmem221	ENSRNOG00000018167	1.00E+08	16	transmembrane protein 221
Niban3	ENSRNOG00000039357	498604	16	niban apoptosis regulator 3
Casp3	ENSRNOG00000010475	25402	16	caspase 3
H1f1	ENSRNOG00000017175	291145	17	H1.1 linker histone, cluster member
Pter	ENSRNOG00000017328	63852	17	phosphotriesterase related
Psmb8	ENSRNOG00000000456	24968	20	proteasome 20S subunit beta 8
Ncr3	ENSRNOG00000000854	294251	20	natural cytotoxicity triggering receptor 3
Cpne5	ENSRNOG00000000522	309650	20	copine 5
Dnmt3l	ENSRNOG00000001212	309680	20	DNA methyltransferase 3 like
Col4a6	ENSRNOG00000056772	363458	X	collagen type IV alpha 6 chain
**Down regulated genes**				
Tmc4	ENSRNOG00000059741	1.04E+08	1	transmembrane channel-like 4
Rps19l2	ENSRNOG00000037897	1.08E+08	1	ribosomal protein S19-like2
Brsk2	ENSRNOG00000020021	NA	1	BR serine/threonine kinase 2
Cyp26a1	ENSRNOG00000016750	154985	1	cytochrome P450, family 26, subfamily a, polypeptide 1
Sv2c	ENSRNOG00000018094	29643	2	synaptic vesicle glycoprotein 2c
Gria2	ENSRNOG00000054204	29627	2	glutamate ionotropic receptor AMPA type subunit 2
Pax8	ENSRNOG00000026203	81819	3	paired box 8
Dpp4	ENSRNOG00000030763	25253	3	dipeptidylpeptidase 4
Nfe2l2	ENSRNOG00000001548	83619	3	nuclear factor, erythroid 2-like 2
Cat	ENSRNOG00000008364	24248	3	catalase
Gss	ENSRNOG00000018964	25458	3	glutathione synthetase
Hnf4a	ENSRNOG00000008895	25735	3	hepatocyte nuclear factor 4, alpha
Sall4	ENSRNOG00000050035	686412	3	spalt-like transcription factor 4

Gene	Ensembl ID	Entrez ID	Chr	Name of the gene
Actg2	ENSRNOG00000029401	25365	4	actin gamma 2, smooth muscle
Slc6a11	ENSRNOG00000005697	79213	4	solute carrier family 6 member 11
Gpr162	ENSRNOG00000016143	362436	4	G protein-coupled receptor 162
LOC690045	ENSRNOG00000061895	690045	4	similar to immunoreceptor Ly49si1
Gprc5a	ENSRNOG00000008412	312790	4	G protein-coupled receptor, class C, group 5, member A
LOC500350	ENSRNOG00000030158	500350	4	LRRGT00139
Etfbkmt	ENSRNOG00000036918	316976	4	electron transfer flavoprotein subunit beta lysine methyltransferase
RGD1564053	ENSRNOG00000005375	500390	5	RIKEN cDNA A830018L16 gene
Aqp3	ENSRNOG00000009797	65133	5	aquaporin 3 (Gill blood group)
Tmem88b	ENSRNOG00000036869	680723	5	transmembrane protein 88B
Vit	ENSRNOG00000004706	313831	6	vitrin
Lhcgr	ENSRNOG00000016712	25477	6	luteinizing hormone/choriogonadotropin receptor
Agr3	ENSRNOG00000004994	298959	6	anterior gradient 3, protein disulphide isomerase
Gpx4	ENSRNOG00000013604	29328	7	glutathione peroxidase 4
Mybpc1	ENSRNOG00000056493	362867	7	myosin binding protein C, slow type
Amdhd1	ENSRNOG00000005266	299735	7	amidohydrolase domain containing 1
Pkhd1l1	ENSRNOG00000004398	314917	7	PKHD1 like 1
Ncf4	ENSRNOG00000006940	500904	7	neutrophil cytosolic factor 4
Sstr3	ENSRNOG00000007332	171044	7	somatostatin receptor 3
Col2a1	ENSRNOG00000058560	25412	7	collagen type II alpha 1 chain
Gpx3	ENSRNOG00000052564	64317	10	glutathione peroxidase 3
Fat2	ENSRNOG00000012575	65048	10	FAT atypical cadherin 2
Sod1	ENSRNOG00000002115	24786	11	superoxide dismutase 1
Scarb1	ENSRNOG00000000981	25073	12	scavenger receptor class B, member 1
Fras1	ENSRNOG00000002053	289486	14	Fraser extracellular matrix complex subunit 1
Ugt2b15	ENSRNOG00000046540	266685	14	UDP glucuronosyltransferase family 2 member B15
Thrb	ENSRNOG00000006649	24831	15	thyroid hormone receptor beta
Rarb	ENSRNOG00000024061	24706	15	retinoic acid receptor, beta
Pnoc	ENSRNOG00000014231	25516	15	prepronociceptin
Syt15	ENSRNOG00000051688	306285	16	synaptotagmin 15
Gsr	ENSRNOG00000014915	116686	16	glutathione-disulfide reductase
Star	ENSRNOG00000015052	25557	16	steroidogenic acute regulatory protein
Hsd17b3	ENSRNOG00000019096	117182	17	hydroxysteroid (17-beta) dehydrogenase 3
Gad2	ENSRNOG00000018200	24380	17	glutamate decarboxylase 2
Cfb	ENSRNOG00000000419	NA	20	complement factor B
Itgb2	ENSRNOG00000001224	309684	20	integrin subunit beta 2
Ar	ENSRNOG00000005639	24208	X	androgen receptor
Slc6a14	ENSRNOG00000005687	298340	X	solute carrier family 6 member 14

Table S6: Summary of deregulated genes in the epididymis of rats exposed to perfluorooctanoic acid during prepubertal period

Up regulated genes

Symbol	Ensembl Gene ID	Entrez	Chr	Description
Vwa3b	ENSRNOG00000023566	501126	9	von Willebrand factor A domain containing 3B
Aldh8a1	ENSRNOG00000014907	685750	1	aldehyde dehydrogenase 8 family, member A1
Ube2ql1	ENSRNOG00000034075	679949	1	ubiquitin-conjugating enzyme E2Q family-like 1
Psg29	ENSRNOG00000029485	292666	1	pregnancy-specific glycoprotein 29
Pinlyp	ENSRNOG00000019862	308429	1	phospholipase A2 inhibitor and LY6/PLAUR domain containing
Zfp575	ENSRNOG00000024065	308430	1	zinc finger protein 575
LOC103689942	ENSRNOG00000050105	287009	1	carcinoembryonic antigen-related cell adhesion molecule 1-like
Cd177	ENSRNOG00000022669	499099	1	CD177 molecule
Cyp2f4	ENSRNOG00000032805	54246	1	cytochrome P450, family 2, subfamily f, polypeptide 4
Rhcg	ENSRNOG00000015526	293048	1	Rh family, C glycoprotein
Nmb	ENSRNOG00000011011	499194	1	neuromedin B
Ap3b2	ENSRNOG00000019249	308777	1	adaptor related protein complex 3 subunit beta 2
Nox4	ENSRNOG00000013925	85431	1	NADPH oxidase 4
Pak1	ENSRNOG00000029784	29431	1	p21 (RAC1) activated kinase 1
Pde2a	ENSRNOG00000019560	81743	1	phosphodiesterase 2A
Folr1	ENSRNOG00000019902	171049	1	folate receptor alpha
Tmc7	ENSRNOG00000016679	499254	1	transmembrane channel-like 7
Cuzd1	ENSRNOG00000029945	117179	1	CUB and zona pellucida-like domains 1
Rplp2	ENSRNOG00000002116	1.01E+08	1	ribosomal protein lateral stalk subunit P2
Ascl2	ENSRNOG00000020434	24209	1	achaete-scute family bHLH transcription factor 2
Rnaseh2c	ENSRNOG00000020700	1E+08	1	ribonuclease H2, subunit C
Cyp26c1	ENSRNOG00000030698	308190	1	cytochrome P450, family 26, subfamily C, polypeptide 1
Cyp26a1	ENSRNOG00000016750	154985	1	cytochrome P450, family 26, subfamily a, polypeptide 1
Tdrd1	ENSRNOG00000017064	292129	1	tudor domain containing 1
Cdh6	ENSRNOG00000013535	25409	2	cadherin 6
Fabp5	ENSRNOG00000049075	140868	2	fatty acid binding protein 5
Stmn2	ENSRNOG00000011705	84510	2	stathmin 2
Veph1	ENSRNOG00000012427	361954	2	ventricular zone expressed PH domain-containing 1
Tchh	ENSRNOG00000056746	310588	2	trichohyalin
Slc6a17	ENSRNOG00000050090	613226	2	solute carrier family 6 member 17
Slc39a8	ENSRNOG00000012508	295455	2	solute carrier family 39 member 8
Clca4	ENSRNOG00000029889	362053	2	chloride channel accessory 4
Tor4a	ENSRNOG00000009369	311795	3	torsin family 4, member A
Barhl1	ENSRNOG00000013209	117232	3	BarH-like homeobox 1
Ntng2	ENSRNOG00000013694	311836	3	netrin G2
Lypd6b	ENSRNOG00000004614	362133	3	LY6/PLAUR domain containing 6B
Chac1	ENSRNOG00000014387	362196	3	ChaC glutathione-specific gamma-glutamylcyclotransferase 1
Mal	ENSRNOG00000015445	25263	3	mal, T-cell differentiation protein
Slx4ip	ENSRNOG00000007430	499895	3	SLX4 interacting protein
Pcsk2	ENSRNOG00000005438	25121	3	proprotein convertase subtilisin/kexin type 2
Defb29	ENSRNOG00000023195	641519	3	defensin beta 29
Defb21	ENSRNOG00000023154	641636	3	defensin beta 21
Spint5p	ENSRNOG00000014807	408232	3	serine peptidase inhibitor, Kunitz type 5
Ripor3	ENSRNOG00000010791	688510	3	RIPOR family member 3
Spo11	ENSRNOG00000006732	366261	3	SPO11, initiator of meiotic double stranded breaks
Reln	ENSRNOG00000021441	24718	4	reelin
Pclo	ENSRNOG00000005726	56768	4	piccolo (presynaptic cytomatrix protein)
Pon3	ENSRNOG00000009096	312086	4	paraoxonase 3
Ccser1	ENSRNOG00000029321	500153	4	coiled-coil serine-rich protein 1
Sftpb	ENSRNOG00000010761	192155	4	surfactant protein B
Slc4a5	ENSRNOG00000010378	297386	4	solute carrier family 4 member 5
Slc6a13	ENSRNOG00000012876	171163	4	solute carrier family 6 member 13
Ly49s7	ENSRNOG00000058775	494203	4	Ly49 stimulatory receptor 7

Symbol	Ensembl Gene ID	Entrez	Chr	Description
Kap	ENSRNOG00000005858	24937	4	kidney androgen regulated protein
RGD1564053	ENSRNOG00000005375	500390	5	RIKEN cDNA A830018L16 gene
Mmp16	ENSRNOG00000005708	65205	5	matrix metallopeptidase 16
Ptgr1	ENSRNOG00000015072	192227	5	prostaglandin reductase 1
Cntln	ENSRNOG00000043151	679640	5	centlein
Cdkn2b	ENSRNOG00000006735	25164	5	cyclin-dependent kinase inhibitor 2B
Pdzk1ip1	ENSRNOG00000008161	81916	5	PDZK1 interacting protein 1
Cyp4a8	ENSRNOG00000008842	266674	5	cytochrome P450, family 4, subfamily a, polypeptide 8
Guca2a	ENSRNOG00000008849	25656	5	guanylate cyclase activator 2A
Rhbdl2	ENSRNOG00000026592	298512	5	rhomboid like 2
Klhdc7a	ENSRNOG00000018867	298590	5	kelch domain containing 7A
Tmem52	ENSRNOG00000016618	1E+08	5	transmembrane protein 52
Tmem178a	ENSRNOG00000007907	362691	6	transmembrane protein 178A
Galnt14	ENSRNOG00000007951	313878	6	polypeptide N-acetylgalactosaminyltransferase 14
Garem2	ENSRNOG00000048004	362801	6	GRB2 associated regulator of MAPK1 subtype 2
Fkbp1b	ENSRNOG00000047143	58950	6	FKBP prolyl isomerase 1B
Cys1	ENSRNOG00000058891	690489	6	cystin 1
Crppa	ENSRNOG00000006199	493574	6	CDP-L-ribitol pyrophosphorylase A
Cdkl1	ENSRNOG00000038720	314198	6	cyclin dependent kinase like 1
Rps6kl1	ENSRNOG00000005530	299202	6	ribosomal protein S6 kinase-like 1
Tshr	ENSRNOG00000003972	25360	6	thyroid stimulating hormone receptor
Clba1	ENSRNOG00000013690	362793	6	clathrin binding box of aftiphilin containing 1
Kcnh3	ENSRNOG00000057315	27150	7	potassium voltage-gated channel subfamily H member 3
Cela1	ENSRNOG00000004725	24331	7	chymotrypsin like elastase 1
Scn8a	ENSRNOG00000005309	29710	7	sodium voltage-gated channel alpha subunit 8
Piwil4	ENSRNOG00000009043	689972	8	piwi-like RNA-mediated gene silencing 4
Fxyd2	ENSRNOG00000016469	29639	8	FXYD domain-containing ion transport regulator 2
Dixdc1	ENSRNOG00000010260	363062	8	DIX domain containing 1
Pstpip1	ENSRNOG00000016413	300732	8	proline-serine-threonine phosphatase-interacting protein 1
Paqr5	ENSRNOG00000014164	315741	8	progestin and adipoQ receptor family member 5
Ubap1l	ENSRNOG00000050979	691572	8	ubiquitin associated protein 1-like
Snx22	ENSRNOG00000022852	300796	8	sorting nexin 22
Gsta5	ENSRNOG00000000201	494499	8	glutathione S-transferase alpha 5
Gsta2	ENSRNOG00000029861	494499	8	glutathione S-transferase alpha 2
Prss35	ENSRNOG00000025184	315866	8	serine protease 35
Celsr3	ENSRNOG00000053889	83466	8	cadherin, EGF LAG seven-pass G-type receptor 3
Spink8	ENSRNOG00000037199	301016	8	serine peptidase inhibitor, Kazal type 8
Dclk3	ENSRNOG00000033026	316023	8	doublecortin-like kinase 3
Topaz1	ENSRNOG00000025601	301075	8	testis and ovary specific PAZ domain containing 1
Mdfi	ENSRNOG00000014987	501097	9	MyoD family inhibitor
Ankrd66	ENSRNOG00000046426	501105	9	ankyrin repeat domain 66
Adgrf4	ENSRNOG00000012535	501106	9	adhesion G protein-coupled receptor F4
Rims1	ENSRNOG00000011000	84556	9	regulating synaptic membrane exocytosis 1
Mefv	ENSRNOG00000008134	58923	10	MEFV innate immuity regulator, pyrin
Shroom1	ENSRNOG00000007431	287285	10	shroom family member 1
Kcnj12	ENSRNOG00000002303	117052	10	potassium inwardly-rectifying channel, subfamily J, member 12
Rnf222	ENSRNOG00000022964	363627	10	ring finger protein 222
Aldoc	ENSRNOG00000011452	24191	10	aldolase, fructose-bisphosphate C
LOC103694863	ENSRNOG00000010302	303382	10	ras-like protein family member 10B
Ac1576	ENSRNOG00000058372	1.03E+08	10	uncharacterized LOC102552783
Ca4	ENSRNOG00000002916	29242	10	carbonic anhydrase 4
Ttll6	ENSRNOG00000004939	287646	10	tubulin tyrosine ligase like 6
Csf3	ENSRNOG00000008525	25610	10	colony stimulating factor 3
Mpp3	ENSRNOG00000033653	114202	10	membrane palmitoylated protein 3
Ace3	ENSRNOG00000007467	498012	10	angiotensin I converting enzyme (peptidyl-dipeptidase A) 3
Galk1	ENSRNOG00000006359	287835	10	galactokinase 1
Nptx1	ENSRNOG00000003741	266777	10	neuronal pentraxin 1

Symbol	Ensembl Gene ID	Entrez	Chr	Description
Fam43a	ENSRNOG00000001728	288031	11	family with sequence similarity 43, member A
newgene_2724	ENSRNOG00000038540	1.08E+08	11	glycoprotein V platelet
Azgp1	ENSRNOG00000001333	25294	12	alpha-2-glycoprotein 1, zinc-binding
Cyb5r1	ENSRNOG00000003973	304805	13	cytochrome b5 reductase 1
Atp6v1g3	ENSRNOG00000022480	289407	13	ATPase H+ transporting V1 subunit G3
RGD1309106	ENSRNOG00000026523	360864	13	similar to hypothetical protein
Spp1	ENSRNOG00000043451	25353	14	secreted phosphoprotein 1
Bmp3	ENSRNOG00000002381	25667	14	bone morphogenetic protein 3
Rassf6	ENSRNOG00000002866	305251	14	Ras association domain family member 6
Gc	ENSRNOG00000003119	24384	14	GC, vitamin D binding protein
Odam	ENSRNOG00000023372	641555	14	odontogenic, ameloblast associated
Fgfbp1	ENSRNOG00000003095	64535	14	fibroblast growth factor binding protein 1
Nefh	ENSRNOG00000008716	24587	14	neurofilament heavy
Cadps	ENSRNOG00000008570	26989	15	calcium dependent secretion activator
Acox2	ENSRNOG00000007378	252898	15	acyl-CoA oxidase 2
Rnase9	ENSRNOG00000029516	364301	15	ribonuclease A family member 9
Ebpl	ENSRNOG00000014659	361054	15	EBP like
Fam167a	ENSRNOG00000011316	498533	15	family with sequence similarity 167, member A
Pde4c	ENSRNOG00000043249	1E+08	16	phosphodiesterase 4C
Aadat	ENSRNOG00000011861	29416	16	aminoadipate aminotransferase
Galnt7	ENSRNOG00000012037	29750	16	polypeptide N-acetylgalactosaminyltransferase 7
Defb13	ENSRNOG00000038157	641626	16	defensin beta 13
Defb39	ENSRNOG00000034275	641646	16	defensin beta 39
Defb12	ENSRNOG00000038155	1.01E+08	16	defensin beta 12
Defb11	ENSRNOG00000038151	641630	16	defensin beta 11
RatNP-3b	ENSRNOG00000038135	498659	16	defensin RatNP-3 precursor
Defb51	ENSRNOG00000038130	641620	16	defensin beta 51
Spag11a	ENSRNOG00000013957	246305	16	sperm associated antigen 11A
Kbtbd11	ENSRNOG00000012333	306617	16	kelch repeat and BTB domain containing 11
Gcnt2	ENSRNOG00000023778	306860	17	glucosaminyl (N-acetyl) transferase 2 (I blood group)
Aoah	ENSRNOG00000054964	498757	17	acyloxyacyl hydrolase
Amph	ENSRNOG00000012490	60668	17	amphiphysin
RGD1564865	ENSRNOG00000038361	498789	17	similar to 20-alpha-hydroxysteroid dehydrogenase
Pkd2l2	ENSRNOG00000025489	291683	18	polycystin 2 like 2, transient receptor potential cation channel
Psd2	ENSRNOG00000019177	307500	18	pleckstrin and Sec7 domain containing 2
Grxcr2	ENSRNOG00000039325	681048	18	glutaredoxin and cysteine rich domain containing 2
Cdo1	ENSRNOG00000000158	81718	18	cysteine dioxygenase type 1
Calb2	ENSRNOG00000016977	117059	19	calbindin 2
Disc1	ENSRNOG00000019779	307940	19	DISC1 scaffold protein
Pou3f1	ENSRNOG00000047686	192110	5	POU class 3 homeobox 1

Down regulated genes

Symbol	Ensembl Gene ID	Entrez	Chr	Description
Arfgef3	ENSRNOG00000011460	292947	1	ARFGEF family member 3
Moxd1	ENSRNOG00000015321	294119	1	monooxygenase, DBH-like 1
Slc18b1	ENSRNOG00000016371	309570	1	solute carrier family 18 member B1
Adcy2	ENSRNOG00000032150	81636	1	adenylate cyclase 2
Smoc2	ENSRNOG00000014166	292401	1	SPARC related modular calcium binding 2
Has1	ENSRNOG00000010994	282821	1	hyaluronan synthase 1
Rnf225	ENSRNOG00000038445	502300	1	ring finger protein 225
Ptprh	ENSRNOG00000058906	171125	1	protein tyrosine phosphatase, receptor type, H
Bsph1	ENSRNOG00000052287	NA	1	binder of sperm protein homolog 1
Fosb	ENSRNOG00000046667	1E+08	1	FosB proto-oncogene, AP-1 transcription factor subunit
Vsig10l	ENSRNOG00000023411	308556	1	V-set and immunoglobulin domain containing 10 like
Clec11a	ENSRNOG00000019138	29313	1	C-type lectin domain containing 11A
RGD1562492	ENSRNOG00000037219	499151	1	Solute carrier family 6 member 16
Hsd17b14	ENSRNOG00000020949	691018	1	hydroxysteroid (17-beta) dehydrogenase 14
Abcc6	ENSRNOG00000028781	81642	1	ATP binding cassette subfamily C member 6
Ptpn5	ENSRNOG00000013981	29644	1	protein tyrosine phosphatase, non-receptor type 5

Symbol	Ensembl Gene ID	Entrez	Chr	Description
Abhd2	ENSRNOG00000017120	293050	1	abhydrolase domain containing 2
RGD1310717	ENSRNOG00000017039	293288	1	similar to RIKEN cDNA E030002O03
Ovch2	ENSRNOG00000019794	308919	1	ovochymase 2
Slc5a11	ENSRNOG00000013407	252854	1	solute carrier family 5 member 11
Lat	ENSRNOG00000017429	81511	1	linker for activation of T cells
Tlcd3b	ENSRNOG00000019914	293493	1	TLC domain containing 3B
Doc2a	ENSRNOG00000019920	65031	1	double C2 domain alpha
LOC308990	ENSRNOG00000016978	308990	1	expressed sequence AI467606
Inpp5f	ENSRNOG00000020388	309008	1	inositol polyphosphate-5-phosphatase F
B4galnt4	ENSRNOG00000053075	309105	1	beta-1,4-N-acetyl-galactosaminyl transferase 4
Ccnd1	ENSRNOG00000020918	58919	1	cyclin D1
Smim38	ENSRNOG00000013373	246306	1	small integral membrane protein 38
Pitpnm1	ENSRNOG00000018553	361694	1	phosphatidylinositol transfer protein, membrane-associated 1
Ptprcap	ENSRNOG00000021724	499300	1	protein tyrosine phosphatase, receptor type, C-associated protein
Syt12	ENSRNOG00000019306	191595	1	synaptotagmin 12
Sptbn2	ENSRNOG00000058842	29211	1	spectrin, beta, non-erythrocytic 2
Gal3st3	ENSRNOG00000028743	499302	1	galactose-3-O-sulfotransferase 3
Ltbp3	ENSRNOG00000020813	83838	1	latent transforming growth factor beta binding protein 3
Dagla	ENSRNOG00000027264	309207	1	diacylglycerol lipase, alpha
Cd6	ENSRNOG00000020884	25752	1	Cd6 molecule
Ptgdr2	ENSRNOG00000036631	309212	1	prostaglandin D2 receptor 2
Ms4a14	ENSRNOG00000025805	293743	1	membrane spanning 4-domains A14
Fam111a	ENSRNOG00000012067	499322	1	family with sequence similarity 111, member A
Glis3	ENSRNOG00000014768	NA	1	GLIS family zinc finger 3
Slc1a1	ENSRNOG00000014816	25550	1	solute carrier family 1 member 1
Cd274	ENSRNOG00000016112	499342	1	CD274 molecule
Rbp4	ENSRNOG00000015518	25703	1	retinol binding protein 4
Lgi1	ENSRNOG00000014758	252892	1	leucine-rich, glioma inactivated 1
Slit1	ENSRNOG00000026065	65047	1	slit guidance ligand 1
Wnt8b	ENSRNOG00000013817	293990	1	Wnt family member 8B
Sec31b	ENSRNOG00000025781	309433	1	SEC31 homolog B, COPII coat complex component
Kazald1	ENSRNOG00000016058	293997	1	Kazal-type serine peptidase inhibitor domain 1
LOC100911951	ENSRNOG00000050450	1.01E+08	1	Kv channel-interacting protein 2-like
Cfap43	ENSRNOG00000036585	365476	1	cilia and flagella associated protein 43
Dusp5	ENSRNOG00000014061	171109	1	dual specificity phosphatase 5
Rbm20	ENSRNOG00000014705	309544	1	RNA binding motif protein 20
Vwa2	ENSRNOG00000025581	307988	1	von Willebrand factor A domain containing 2
Hapln1	ENSRNOG00000032002	29331	2	hyaluronan and proteoglycan link protein 1
Vcan	ENSRNOG00000029212	114122	2	versican
Gcnt4	ENSRNOG00000055451	NA	2	glucosaminyl (N-acetyl) transferase 4
LOC108348103	ENSRNOG00000045693	1.01E+08	2	serine protease inhibitor Kazal-type 5-like
Il31ra	ENSRNOG00000042080	688622	2	interleukin 31 receptor A
Marchf11	ENSRNOG00000024461	499558	2	membrane associated ring-CH-type finger 11
Ca3	ENSRNOG00000010079	54232	2	carbonic anhydrase 3
Pcsk1	ENSRNOG00000011107	25204	2	proprotein convertase subtilisin/kexin type 1
tGap1	ENSRNOG00000022511	294892	2	GTPase activating protein testicular GAP1
Skil	ENSRNOG00000009899	114208	2	SKI-like proto-oncogene
Qrfpr	ENSRNOG00000014414	310327	2	pyroglutamylated RFamide peptide receptor
Mgst2	ENSRNOG00000061857	295037	2	microsomal glutathione S-transferase 2
Mme	ENSRNOG00000009514	24590	2	membrane metallo-endopeptidase
Arl14	ENSRNOG00000049827	1.04E+08	2	ADP-ribosylation factor like GTPase 14
B3galnt1	ENSRNOG00000012019	310508	2	beta-1,3-N-acetylgalactosaminyltransferase 1 (globoside blood group)
Sfrp2	ENSRNOG00000009465	310552	2	secreted frizzled-related protein 2
Tmem154	ENSRNOG00000010866	361972	2	transmembrane protein 154
Sema4a	ENSRNOG00000019737	310630	2	semaphorin 4A
Trim46	ENSRNOG00000055433	310641	2	tripartite motif-containing 46
RGD1560554	ENSRNOG00000060494	502576	2	similar to TDPOZ2
C2cd4d	ENSRNOG00000020832	1E+08	2	C2 calcium-dependent domain containing 4D
Tdrkh	ENSRNOG00000020860	310652	2	tudor and KH domain containing
Mllt11	ENSRNOG00000021110	295264	2	MLLT11, transcription factor 7 cofactor
Bnipl	ENSRNOG00000021116	361994	2	BCL2 interacting protein like
Anxa9	ENSRNOG00000021134	689830	2	annexin A9
Gja5	ENSRNOG00000017484	50563	2	gap junction protein, alpha 5
Lef1	ENSRNOG00000010121	161452	2	lymphoid enhancer binding factor 1
Dkk2	ENSRNOG00000011360	295445	2	dickkopf WNT signaling pathway inhibitor 2
LOC102556447	ENSRNOG00000036958	1.03E+08	2	rho guanine nucleotide exchange factor 38-like
Kyat3	ENSRNOG00000043426	541589	2	kynurenine aminotransferase 3

Symbol	Ensembl Gene ID	Entrez	Chr	Description
Ccn1	ENSRNOG00000014350	83476	2	cellular communication network factor 1
Dnase2b	ENSRNOG00000016262	59296	2	deoxyribonuclease 2 beta
Uox	ENSRNOG00000016339	114768	2	urate oxidase
Lcn8	ENSRNOG00000017161	366008	3	lipocalin 8
Lcn6	ENSRNOG00000028095	414138	3	lipocalin 6
Lcn10	ENSRNOG00000028074	499756	3	lipocalin 10
Obp2a	ENSRNOG00000042407	680754	3	odorant binding protein 2A
Lcn9	ENSRNOG00000027821	296578	3	lipocalin 9
Agpat2	ENSRNOG00000019466	311821	3	1-acylglycerol-3-phosphate O-acyltransferase 2
Olfm1	ENSRNOG00000009862	93667	3	olfactomedin 1
Lcn2	ENSRNOG00000013973	170496	3	lipocalin 2
St6galnac4	ENSRNOG00000048870	407764	3	ST6 N-acetylgalactosaminide alpha-2,6-sialyltransferase 4
Olfml2a	ENSRNOG00000014034	296708	3	olfactomedin-like 2A
Kcnj3	ENSRNOG00000005369	50599	3	potassium inwardly-rectifying channel, subfamily J, member 3
Cers6	ENSRNOG00000024595	366065	3	ceramide synthase 6
Gpr155	ENSRNOG00000018485	311730	3	G protein-coupled receptor 155
Hoxd10	ENSRNOG00000001581	303991	3	homeo box D10
Frzb	ENSRNOG00000007765	295691	3	frizzled-related protein
P2rx3	ENSRNOG00000008552	81739	3	purinergic receptor P2X 3
Lrp4	ENSRNOG00000015285	83469	3	LDL receptor related protein 4
Tspan18	ENSRNOG00000008758	311210	3	tetraspanin 18
Rcn1	ENSRNOG00000013452	362182	3	reticulocalbin 1
Muc15	ENSRNOG00000004703	690914	3	mucin 15, cell surface associated
Ryr3	ENSRNOG00000006645	170546	3	ryanodine receptor 3
Tyro3	ENSRNOG00000058586	25232	3	TYRO3 protein tyrosine kinase
Ell3	ENSRNOG00000022868	296102	3	elongation factor for RNA polymerase II 3
Frmd5	ENSRNOG00000016189	311362	3	FERM domain containing 5
Sema6d	ENSRNOG00000004812	311384	3	semaphorin 6D
Shc4	ENSRNOG00000037134	679845	3	SHC adaptor protein 4
Prom2	ENSRNOG00000014710	192211	3	prominin 2
Ebf4	ENSRNOG00000007408	680751	3	EBF family member 4
Adra1d	ENSRNOG00000021256	29413	3	adrenoceptor alpha 1D
Prnp	ENSRNOG00000021259	24686	3	prion protein
Cst11	ENSRNOG00000004808	245916	3	cystatin 11
Cst12	ENSRNOG00000004946	266776	3	cystatin 12
Cst8	ENSRNOG00000004989	29679	3	cystatin 8
LOC689081	ENSRNOG00000005178	689081	3	similar to cystatin E2
Ninl	ENSRNOG00000027747	311529	3	ninein-like
Rad21l1	ENSRNOG00000022335	689823	3	RAD21 cohesin complex component like 1
Slc52a3	ENSRNOG00000005132	311536	3	solute carrier family 52 member 3
Defb20	ENSRNOG00000023384	641641	3	defensin beta 20
Defb27	ENSRNOG00000036896	641642	3	defensin beta 27
Defb25	ENSRNOG00000036895	641644	3	defensin beta 25
LOC690507	ENSRNOG00000015637	690507	3	similar to Vomeromodulin
Soga1	ENSRNOG00000053240	311578	3	suppressor of glucose, autophagy associated 1
Nnat	ENSRNOG00000024923	94270	3	neuronatin
RGD1563354	ENSRNOG00000014424	311592	3	RIKEN cDNA D630003M21 gene
Bpi	ENSRNOG00000034195	296321	3	bactericidal/permeability-increasing protein
Lbp	ENSRNOG00000014532	29469	3	lipopolysaccharide binding protein
Ptprt	ENSRNOG00000032656	362263	3	protein tyrosine phosphatase, receptor type, T
Hnf4a	ENSRNOG00000008895	25735	3	hepatocyte nuclear factor 4, alpha
Eppin	ENSRNOG00000028908	685161	3	epididymal peptidase inhibitor
Pmepa1	ENSRNOG00000050404	311676	3	prostate transmembrane protein, androgen induced 1
Tcfl5	ENSRNOG00000061199	311715	3	transcription factor like 5
Gsap	ENSRNOG00000028801	311984	4	gamma-secretase activating protein
Cftr	ENSRNOG00000055103	24255	4	CF transmembrane conductance regulator
Cttnbp2	ENSRNOG00000061845	282587	4	cortactin binding protein 2
Tcaf1	ENSRNOG00000017911	362353	4	TRPM8 channel-associated factor 1
Sspo	ENSRNOG00000025848	474348	4	SCO-spondin
LOC100911668	ENSRNOG00000050252	1.01E+08	4	homeobox protein Hox-A10-like
Chn2	ENSRNOG00000009411	84031	4	chimerin 2
Inmt	ENSRNOG00000011250	368066	4	indolethylamine N-methyltransferase
Aqp1	ENSRNOG00000011648	25240	4	aquaporin 1 (Colton blood group)
Vopp1	ENSRNOG00000006646	362374	4	VOPP1 WW domain binding protein
Tacstd2	ENSRNOG00000007740	494343	4	tumor-associated calcium signal transducer 2
Efcc1	ENSRNOG00000009787	685202	4	EF-hand and coiled-coil domain containing 1
Slc41a3	ENSRNOG00000045821	641603	4	solute carrier family 41, member 3

Symbol	Ensembl Gene ID	Entrez	Chr	Description
Cidec	ENSRNOG00000009153	500292	4	cell death-inducing DFFA-like effector c
Ret	ENSRNOG00000014751	24716	4	ret proto-oncogene
B4galnt3	ENSRNOG00000010344	500306	4	beta-1,4-N-acetyl-galactosaminyl transferase 3
Cecr2	ENSRNOG00000011566	500308	4	CECR2, histone acetyl-lysine reader
Ntf3	ENSRNOG00000019716	81737	4	neurotrophin 3
Kcna1	ENSRNOG00000019750	24520	4	potassium voltage-gated channel subfamily A member 1
Gys2	ENSRNOG00000059753	25623	4	glycogen synthase 2
Cnr1	ENSRNOG00000008223	25248	5	cannabinoid receptor 1
Dnajb5	ENSRNOG00000000130	313811	5	DnaJ heat shock protein family (Hsp40) member B5
Aldob	ENSRNOG00000006807	24190	5	aldolase, fructose-bisphosphate B
Musk	ENSRNOG00000033567	81725	5	muscle associated receptor tyrosine kinase
Orm1	ENSRNOG00000007886	24614	5	orosomucoid 1
Faah	ENSRNOG00000045949	1.01E+08	5	fatty acid amide hydrolase
Slc6a9	ENSRNOG00000019484	116509	5	solute carrier family 6 member 9
Mfsd2a	ENSRNOG00000014008	298504	5	major facilitator superfamily domain containing 2A
Heyl	ENSRNOG00000015318	313575	5	hes-related family bHLH transcription factor with YRPW motif-like
Cd164l2	ENSRNOG00000009505	1E+08	5	CD164 molecule like 2
Slc30a2	ENSRNOG00000054142	25362	5	solute carrier family 30 member 2
Ncmap	ENSRNOG00000048139	689826	5	noncompact myelin associated protein
Rnf186	ENSRNOG00000017244	690433	5	ring finger protein 186
Fblim1	ENSRNOG00000011774	362650	5	filamin binding LIM protein 1
Prdm16	ENSRNOG00000045913	1E+08	5	PR/SET domain 16
C1qtnf12	ENSRNOG00000019864	313774	5	C1q and TNF related 12
Qpct	ENSRNOG00000005705	313837	6	glutaminyl-peptide cyclotransferase
Kcng3	ENSRNOG00000004535	171011	6	potassium voltage-gated channel modifier subfamily G member 3
Haao	ENSRNOG00000031263	56823	6	3-hydroxyanthranilate 3,4-dioxygenase
Alk	ENSRNOG00000008683	266802	6	ALK receptor tyrosine kinase
Togaram2	ENSRNOG00000026344	313892	6	TOG array regulator of axonemal microtubules 2
Otof	ENSRNOG00000009967	84573	6	otoferlin
Mycn	ENSRNOG00000051372	298894	6	MYCN proto-oncogene, bHLH transcription factor
Greb1	ENSRNOG00000024651	500633	6	growth regulating estrogen receptor binding 1
Etv1	ENSRNOG00000006867	362733	6	ETS variant transcription factor 1
Egln3	ENSRNOG00000005053	54702	6	egl-9 family hypoxia-inducible factor 3
Sstr1	ENSRNOG00000048145	25033	6	somatostatin receptor 1
Six4	ENSRNOG00000007250	299138	6	SIX homeobox 4
Plek2	ENSRNOG00000010098	314260	6	pleckstrin 2
Papln	ENSRNOG00000009448	314297	6	papilin, proteoglycan-like sulfated glycoprotein
Fos	ENSRNOG00000008015	314322	6	Fos proto-oncogene, AP-1 transcription factor subunit
Kcnk13	ENSRNOG00000047363	64120	6	potassium two pore domain channel subfamily K member 13
Slc24a4	ENSRNOG00000006729	314396	6	solute carrier family 24 member 4
Serpina16	ENSRNOG00000026297	299271	6	serine (or cysteine) peptidase inhibitor, clade A
Slc25a47	ENSRNOG00000003020	299316	6	solute carrier family 25, member 47
Tmem121	ENSRNOG00000005174	691678	6	transmembrane protein 121
Igh-6	ENSRNOG00000048402	NA	6	immunoglobulin heavy chain 6
Ighg1	ENSRNOG00000030332	NA	6	immunoglobulin heavy constant gamma 1
RGD1562420	ENSRNOG00000005826	NA	6	similar to hypothetical protein
Pros1	ENSRNOG00000048723	81750	7	protein S
Dgka	ENSRNOG00000022943	140866	7	diacylglycerol kinase, alpha
Gdf11	ENSRNOG00000007610	29454	7	growth differentiation factor 11
Cd63	ENSRNOG00000007650	29186	7	Cd63 molecule
Cbarp	ENSRNOG00000024349	314622	7	CACN subunit beta associated regulatory protein
Prtn3	ENSRNOG00000029814	314615	7	proteinase 3
Plppr3	ENSRNOG00000027940	314614	7	phospholipid phosphatase related 3
Slc41a2	ENSRNOG00000008713	362861	7	solute carrier family 41 member 2
Anks1b	ENSRNOG00000024870	314721	7	ankyrin repeat and sterile alpha motif domain containing 1B
Dusp6	ENSRNOG00000023896	116663	7	dual specificity phosphatase 6
RGD1561648	ENSRNOG00000023824	500841	7	RGD1561648
Sdr9c7	ENSRNOG00000004459	259235	7	short chain dehydrogenase/reductase family 9C, member 7
Enpp2	ENSRNOG00000004089	84050	7	ectonucleotide pyrophosphatase/phosphodiesterase 2
Sntb1	ENSRNOG00000004821	299940	7	syntrophin, beta 1
Ndrg1	ENSRNOG00000007393	299923	7	N-myc downstream regulated 1
Arc	ENSRNOG00000043465	54323	7	activity-regulated cytoskeleton-associated protein
LOC680875	ENSRNOG00000023828	680875	7	similar to dystonin isoform 1
Scx	ENSRNOG00000021812	680712	7	scleraxis bHLH transcription factor
Syngr1	ENSRNOG00000017108	29205	7	synaptogyrin 1
Tnrc6b	ENSRNOG00000024907	192178	7	trinucleotide repeat containing adaptor 6B
Csdc2	ENSRNOG00000005332	266600	7	cold shock domain containing C2

Symbol	Ensembl Gene ID	Entrez	Chr	Description
Cyp2d2	ENSRNOG00000008988	25053	7	cytochrome P450, family 2, subfamily d, polypeptide 2
Efcab6	ENSRNOG00000011094	315179	7	EF-hand calcium binding domain 6
Pnpla3	ENSRNOG00000022268	362972	7	patatin-like phospholipase domain containing 3
Wnt7b	ENSRNOG00000015750	315196	7	Wnt family member 7B
Celsr1	ENSRNOG00000021285	300128	7	cadherin, EGF LAG seven-pass G-type receptor 1
Aqp2	ENSRNOG00000054378	25386	7	aquaporin 2
Nr4a1	ENSRNOG00000007607	79240	7	nuclear receptor subfamily 4, group A, member 1
Krt79	ENSRNOG00000058340	683720	7	keratin 79
Pde1b	ENSRNOG00000036828	29691	7	phosphodiesterase 1B
Ppp1r1a	ENSRNOG00000036827	58977	7	protein phosphatase 1, regulatory (inhibitor) subunit 1A
Mmp7	ENSRNOG00000010507	25335	8	matrix metallopeptidase 7
Gpr83	ENSRNOG00000030318	140595	8	G protein-coupled receptor 83
Kirrel3	ENSRNOG00000009772	315546	8	kirre like nephrin family adhesion molecule 3
St3gal4	ENSRNOG00000009850	363040	8	ST3 beta-galactoside alpha-2,3-sialyltransferase 4
Pate1	ENSRNOG00000049600	1E+08	8	prostate and testis expressed 1
LOC100359924	ENSRNOG00000049218	1E+08	8	prostate and testis expressed N-like
RGD1304554	ENSRNOG00000042925	363043	8	LOC363043
LOC100362078	ENSRNOG00000024026	1E+08	8	prostate and testis expressed family member-like
Pate-f	ENSRNOG00000049588	1.01E+08	8	prostate and testis expressed protein F
Tecta	ENSRNOG00000031126	300653	8	tectorin alpha
Abcg4	ENSRNOG00000008862	300664	8	ATP binding cassette subfamily G member 4
Sidt2	ENSRNOG00000017871	315617	8	SID1 transmembrane family, member 2
Pou2af1	ENSRNOG00000011500	690528	8	POU class 2 homeobox associating factor 1
Stra6	ENSRNOG00000008312	363071	8	stimulated by retinoic acid 6
Loxl1	ENSRNOG00000008680	315714	8	lysyl oxidase-like 1
Aqp9	ENSRNOG00000061883	65054	8	aquaporin 9
Cd109	ENSRNOG00000025332	363104	8	CD109 molecule
Elovl4	ENSRNOG00000009773	315851	8	ELOVL fatty acid elongase 4
Tmed3	ENSRNOG00000013889	300888	8	transmembrane p24 trafficking protein 3
Rasgrf1	ENSRNOG00000014025	192213	8	RAS protein-specific guanine nucleotide-releasing factor 1
Adamts7	ENSRNOG00000028036	315879	8	ADAM metallopeptidase with thrombospondin type 1 motif, 7
Plod2	ENSRNOG00000030183	300901	8	procollagen lysine, 2-oxoglutarate 5-dioxygenase 2
Rbp1	ENSRNOG00000013794	25056	8	retinol binding protein 1
Ky	ENSRNOG00000008210	315962	8	kyphoscoliosis peptidase
Sema3b	ENSRNOG00000016512	363142	8	semaphorin 3B
Slc22a13	ENSRNOG00000056476	316062	8	solute carrier family 22 member 13
LOC685081	ENSRNOG00000042660	685081	8	similar to solute carrier family 22
C3	ENSRNOG00000046834	24232	9	complement C3
Chaf1a	ENSRNOG00000046479	363333	9	chromatin assembly factor 1 subunit A
Runx2	ENSRNOG00000020193	367218	9	RUNX family transcription factor 2
Defb18	ENSRNOG00000039650	641655	9	defensin beta 18
Inpp4a	ENSRNOG00000017660	80849	9	inositol polyphosphate-4-phosphatase type I A
Dnah7	ENSRNOG00000060984	252893	9	dynein, axonemal, heavy chain 7
Raph1	ENSRNOG00000014722	363239	9	Ras association (RalGDS/AF-6) and pleckstrin homology domains 1
Cfap65	ENSRNOG00000056996	301521	9	cilia and flagella associated protein 65
Mogat1	ENSRNOG00000014692	363261	9	monoacylglycerol O-acyltransferase 1
Slc16a14	ENSRNOG00000017072	316578	9	solute carrier family 16, member 14
LOC100362216	ENSRNOG00000017557	1E+08	9	RIKEN cDNA 2810459M11 gene
Alpg	ENSRNOG00000042889	367308	9	alkaline phosphatase, germ cell
Ecel1	ENSRNOG00000019447	60417	9	endothelin converting enzyme-like 1
Inpp5d	ENSRNOG00000017020	54259	9	inositol polyphosphate-5-phosphatase D
Mroh2a	ENSRNOG00000042182	301596	9	maestro heat-like repeat family member 2A
Efna5	ENSRNOG00000034177	116683	9	ephrin A5
Pam	ENSRNOG00000033280	25508	9	peptidylglycine alpha-amidating monooxygenase
Litaf	ENSRNOG00000002520	65161	10	lipopolysaccharide-induced TNF factor
Tnp2	ENSRNOG00000002566	24840	10	transition protein 2
Abat	ENSRNOG00000002636	81632	10	4-aminobutyrate aminotransferase
Hba-a1	ENSRNOG00000029886	25632	10	hemoglobin alpha, adult chain 1
Nipal4	ENSRNOG00000006255	303070	10	NIPA-like domain containing 4
Flt4	ENSRNOG00000002511	114110	10	fms-related tyrosine kinase 4
Slc36a1	ENSRNOG00000012356	155205	10	solute carrier family 36 member 1
Alox15b	ENSRNOG00000007778	266604	10	arachidonate 15-lipoxygenase, type B
Spns2	ENSRNOG00000057040	1E+08	10	sphingolipid transporter 2
Atp2a3	ENSRNOG00000017912	25391	10	ATPase sarcoplasmic/endoplasmic reticulum Ca2+ transporting 3
P2rx1	ENSRNOG00000017606	25505	10	purinergic receptor P2X 1
Camkk1	ENSRNOG00000018242	60341	10	calcium/calmodulin-dependent protein kinase kinase 1
Serpinf1	ENSRNOG00000003172	287526	10	serpin family F member 1

Symbol	Ensembl Gene ID	Entrez	Chr	Description
Serpinf2	ENSRNOG00000003233	287527	10	serpin family F member 2
Tlcd2	ENSRNOG00000037275	497955	10	TLC domain containing 2
Slc13a2	ENSRNOG00000010337	65202	10	solute carrier family 13 member 2
Tac4	ENSRNOG00000004404	282829	10	tachykinin precursor 4
Cacnb1	ENSRNOG00000004518	50688	10	calcium voltage-gated channel auxiliary subunit beta 1
Ppp1r1b	ENSRNOG00000028404	360616	10	protein phosphatase 1, regulatory (inhibitor) subunit 1B
Pnmt	ENSRNOG00000046057	24661	10	phenylethanolamine-N-methyltransferase
Kcnh4	ENSRNOG00000018790	114032	10	potassium voltage-gated channel subfamily H member 4
Etv4	ENSRNOG00000020792	360635	10	ETS variant transcription factor 4
Sost	ENSRNOG00000020805	80722	10	sclerostin
Nags	ENSRNOG00000020879	303563	10	N-acetylglutamate synthase
Mrc2	ENSRNOG00000006548	498011	10	mannose receptor, C type 2
Kif19	ENSRNOG00000003105	303659	10	kinesin family member 19
Rhbdf2	ENSRNOG00000011459	303690	10	rhomboid 5 homolog 2
St6galnac1	ENSRNOG00000000251	287920	10	ST6 N-acetylgalactosaminide alpha-2,6-sialyltransferase 1
Engase	ENSRNOG00000027498	303702	10	endo-beta-N-acetylglucosaminidase
Pycr1	ENSRNOG00000036682	287877	10	pyrroline-5-carboxylate reductase 1
Lipi	ENSRNOG00000033441	288322	11	lipase I
Runx1	ENSRNOG00000001704	50662	11	RUNX family transcription factor 1
Cldn14	ENSRNOG00000001691	304073	11	claudin 14
Sim2	ENSRNOG00000054203	304071	11	SIM bHLH transcription factor 2
Pcp4	ENSRNOG00000001628	25510	11	Purkinje cell protein 4
Nectin3	ENSRNOG00000002176	288124	11	nectin cell adhesion molecule 3
LOC685680	ENSRNOG00000022136	685680	11	similar to TPA-induced transmembrane protein
Cfap44	ENSRNOG00000028077	363782	11	cilia and flagella associated protein 44
Sidt1	ENSRNOG00000002013	288109	11	SID1 transmembrane family, member 1
Igsf11	ENSRNOG00000001525	303926	11	immunoglobulin superfamily, member 11
B4galt4	ENSRNOG00000003114	303923	11	beta-1,4-galactosyltransferase 4
Sema5b	ENSRNOG00000002238	303901	11	semaphorin 5B
Slc12a8	ENSRNOG00000001792	266733	11	solute carrier family 12, member 8
Atp13a4	ENSRNOG00000001714	288026	11	ATPase 13A4
Plaat1	ENSRNOG00000001711	288025	11	phospholipase A and acyltransferase 1
St6gal1	ENSRNOG00000001823	25197	11	ST6 beta-galactoside alpha-2,6-sialyltransferase 1
Adipoq	ENSRNOG00000001821	246253	11	adiponectin, C1Q and collagen domain containing
Etv5	ENSRNOG00000001785	303828	11	ETS variant transcription factor 5
Retn	ENSRNOG00000001001	246250	12	resistin
Nptx2	ENSRNOG00000001006	288475	12	neuronal pentraxin 2
Bhlha15	ENSRNOG00000025164	25334	12	basic helix-loop-helix family, member a15
Stx1a	ENSRNOG00000029165	116470	12	syntaxin 1A
Rimbp2	ENSRNOG00000022893	266780	12	RIMS binding protein 2
Rilpl1	ENSRNOG00000001055	304469	12	Rab interacting lysosomal protein-like 1
Tesc	ENSRNOG00000001128	288689	12	tescalcin
Nos1	ENSRNOG00000001130	24598	12	nitric oxide synthase 1
Acacb	ENSRNOG00000000658	116719	12	acetyl-CoA carboxylase beta
Tpst2	ENSRNOG00000000664	288719	12	tyrosylprotein sulfotransferase 2
Galnt9	ENSRNOG00000037476	304571	12	polypeptide N-acetylgalactosaminyltransferase 9
Lrcol1	ENSRNOG00000037466	1E+08	12	leucine rich colipase-like 1
Serpinb8	ENSRNOG00000002396	288937	13	serpin family B member 8
Sctr	ENSRNOG00000049766	81779	13	secretin receptor
Mgat5	ENSRNOG00000003614	65271	13	alpha-1,6-mannosylglycoprotein 6-beta-N-acetylglucosaminyltransferase
Cd55	ENSRNOG00000003927	64036	13	CD55 molecule (Cromer blood group)
Nfasc	ENSRNOG00000030515	116690	13	neurofascin
Tnni1	ENSRNOG00000009073	29388	13	troponin I1, slow skeletal type
Aspm	ENSRNOG00000012318	289054	13	abnormal spindle microtubule assembly
Ptgs2	ENSRNOG00000002525	29527	13	prostaglandin-endoperoxide synthase 2
LOC684709	ENSRNOG00000046786	684709	13	similar to putative membrane protein Re9
Tstd1	ENSRNOG00000047123	1E+08	13	thiosulfate sulfurtransferase like domain containing 1
Slamf7	ENSRNOG00000023209	364049	13	SLAM family member 7
Vangl2	ENSRNOG00000004889	289229	13	VANGL planar cell polarity protein 2
Igsf9	ENSRNOG00000008054	304982	13	immunoglobulin superfamily, member 9
Capn8	ENSRNOG00000003468	170808	13	calpain 8
Susd4	ENSRNOG00000003562	289335	13	sushi domain containing 4
Cd34	ENSRNOG00000045558	305081	13	CD34 molecule
Lrrc8c	ENSRNOG00000002122	289443	14	leucine rich repeat containing 8 VRAC subunit C
Tmem150c	ENSRNOG00000002258	360916	14	transmembrane protein 150C
Fras1	ENSRNOG00000002053	289486	14	Fraser extracellular matrix complex subunit 1

Symbol	Ensembl Gene ID	Entrez	Chr	Description
Slc4a4	ENSRNOG00000003134	84484	14	solute carrier family 4 member 4
Adgrl3	ENSRNOG00000030149	170641	14	adhesion G protein-coupled receptor L3
Uchl1	ENSRNOG00000002343	29545	14	ubiquitin C-terminal hydrolase L1
Rell1	ENSRNOG00000002192	289635	14	RELT-like 1
Nsg1	ENSRNOG00000005700	25247	14	neuronal vesicle trafficking associated 1
Sh3tc1	ENSRNOG00000007993	305441	14	SH3 domain and tetratricopeptide repeats 1
Hgfac	ENSRNOG00000009572	58947	14	HGF activator
Cfap99	ENSRNOG00000058434	305453	14	cilia and flagella associated protein 99
Slc5a1	ENSRNOG00000017775	25552	14	solute carrier family 5 member 1
Selenom	ENSRNOG00000061231	498398	14	selenoprotein M
Sec14l4	ENSRNOG00000004555	498399	14	SEC14-like lipid binding 4
Camk2b	ENSRNOG00000052080	24245	14	calcium/calmodulin-dependent protein kinase II beta
Adcy1	ENSRNOG00000059479	305509	14	adenylate cyclase 1
Ptger2	ENSRNOG00000050968	81752	15	prostaglandin E receptor 2
Gch1	ENSRNOG00000011039	29244	15	GTP cyclohydrolase 1
Rnase10	ENSRNOG00000010261	305840	15	ribonuclease A family member 10
Rnase13	ENSRNOG00000039500	497194	15	ribonuclease A family member 13
Sall2	ENSRNOG00000013287	305854	15	spalt-like transcription factor 2
Gjb2	ENSRNOG00000008855	394266	15	gap junction protein, beta 2
Ephx2	ENSRNOG00000017286	65030	15	epoxide hydrolase 2
Adra1a	ENSRNOG00000009522	29412	15	adrenoceptor alpha 1A
Defb42	ENSRNOG00000038762	641657	15	defensin beta 42
Defb41	ENSRNOG00000038760	641650	15	defensin beta 41
Fzd3	ENSRNOG00000047211	266715	15	frizzled class receptor 3
Adamdec1	ENSRNOG00000030724	290338	15	ADAM-like, decysin 1
Adam28	ENSRNOG00000014518	290344	15	ADAM metallopeptidase domain 28
Slitrk6	ENSRNOG00000022337	290467	15	SLIT and NTRK-like family, member 6
Abcc4	ENSRNOG00000010064	170924	15	ATP binding cassette subfamily C member 4
Cldn10	ENSRNOG00000010085	290485	15	claudin 10
Dnah1	ENSRNOG00000026914	171339	16	dynein, axonemal, heavy chain 1
Lpl	ENSRNOG00000012181	24539	16	lipoprotein lipase
Pdgfrl	ENSRNOG00000010832	290771	16	platelet-derived growth factor receptor-like
Adam5	ENSRNOG00000017518	498654	16	ADAM metallopeptidase domain 5
Spag11bl	ENSRNOG00000031111	1E+08	16	sperm associated antigen 11b-like
Fgfr4	ENSRNOG00000016763	25114	17	fibroblast growth factor receptor 4
Ror2	ENSRNOG00000053232	306782	17	receptor tyrosine kinase-like orphan receptor 2
Id4	ENSRNOG00000016099	291023	17	inhibitor of DNA binding 4, HLH protein
Atxn1	ENSRNOG00000016998	25049	17	ataxin 1
Cmahp	ENSRNOG00000003094	361245	17	cytidine monophospho-N-acetylneuraminic acid hydroxylase,
Inhba	ENSRNOG00000014320	29200	17	inhibin subunit beta A
Jcad	ENSRNOG00000027938	498764	17	junctional cadherin 5 associated
Chrm3	ENSRNOG00000049410	24260	17	cholinergic receptor, muscarinic 3
Ryr2	ENSRNOG00000017060	689560	17	ryanodine receptor 2
Rnf125	ENSRNOG00000057832	361296	18	ring finger protein 125
Myo7b	ENSRNOG00000015035	498834	18	myosin VIIb
Hbegf	ENSRNOG00000018646	25433	18	heparin-binding EGF-like growth factor
Pcdhgc3	ENSRNOG00000019799	NA	18	protocadherin gamma subfamily C, 3
Fgf1	ENSRNOG00000013867	25317	18	fibroblast growth factor 1
Spink5	ENSRNOG00000013116	361319	18	serine peptidase inhibitor, Kazal type 5
LOC100911797	ENSRNOG00000050548	1.01E+08	18	serine protease inhibitor Kazal-type 5-like
Sema6a	ENSRNOG00000004033	361324	18	semaphorin 6A
Minar2	ENSRNOG00000022429	291580	18	membrane integral NOTCH2 associated receptor 2
Chsy3	ENSRNOG00000050152	291577	18	chondroitin sulfate synthase 3
Synpo	ENSRNOG00000019181	60324	18	synaptopodin
Ablim3	ENSRNOG00000019365	307395	18	actin binding LIM protein family, member 3
LOC679149	ENSRNOG00000048039	679149	19	similar to carboxylesterase 2 (intestine, liver)
LOC102554842	ENSRNOG00000057998	1.03E+08	19	CKLF-like MARVEL transmembrane domain-containing protein 2Bl
Cmtm2a	ENSRNOG00000025420	307616	19	CKLF-like MARVEL transmembrane domain containing 2A
Bean1	ENSRNOG00000013301	361358	19	brain expressed, associated with NEDD4, 1
Slc6a2	ENSRNOG00000016311	83511	19	solute carrier family 6 member 2
Ces4a	ENSRNOG00000014257	291955	19	carboxylesterase 4A
Tmed6	ENSRNOG00000020406	291991	19	transmembrane p24 trafficking protein 6
Dynlrb2	ENSRNOG00000012450	361415	19	dynein light chain roadblock-type 2
Cmip	ENSRNOG00000013178	292051	19	c-Maf-inducing protein
Slc38a8	ENSRNOG00000025503	502208	19	solute carrier family 38, member 8
Atp2c2	ENSRNOG00000049334	171496	19	ATPase secretory pathway Ca2+ transporting 2
Agt	ENSRNOG00000018445	24179	19	angiotensinogen

Symbol	Ensembl Gene ID	Entrez	Chr	Description
Ppp1r18	ENSRNOG00000000816	361790	20	protein phosphatase 1, regulatory subunit 18
Ly6g5c	ENSRNOG00000000846	294245	20	lymphocyte antigen 6 family member G5C
Ly6g5b	ENSRNOG00000027516	406867	20	lymphocyte antigen 6 family member G5B
Ftcd	ENSRNOG00000001261	89833	20	formimidoyltransferase cyclodeaminase
Derl3	ENSRNOG00000028243	690315	20	derlin 3
Ggt1	ENSRNOG00000047697	116568	20	gamma-glutamyltransferase 1
Egr2	ENSRNOG00000000640	114090	20	early growth response 2
Spock2	ENSRNOG00000061544	361840	20	SPARC/osteonectin, cwcv and kazal like domains proteoglycan 2
Unc5b	ENSRNOG00000000567	60630	20	unc-5 netrin receptor B
Pcbd1	ENSRNOG00000000566	29700	20	pterin-4 alpha-carbinolamine dehydratase 1
Fam184a	ENSRNOG00000026407	361853	20	family with sequence similarity 184, member A
Mical1	ENSRNOG00000000307	294520	20	microtubule associated monooxygenase, calponin and LIM domain 1
Uba1y	ENSRNOG00000052326	25225	Y	ubiquitin-activating enzyme, Chr Y
Efhc2	ENSRNOG00000002986	302507	X	EF-hand domain containing 2
Slc38a5	ENSRNOG00000027767	192208	X	solute carrier family 38, member 5
Was	ENSRNOG00000031058	317371	X	WASP actin nucleation promoting factor
Clcn5	ENSRNOG00000002862	25749	X	chloride voltage-gated channel 5
LOC681355	ENSRNOG00000039063	681355	X	similar to potassium channel tetramerisation domain containing 12b
Cltrn	ENSRNOG00000003960	57395	X	collectrin, amino acid transport regulator
Smpx	ENSRNOG00000007495	84416	X	small muscle protein, X-linked
Phex	ENSRNOG00000023440	25512	X	phosphate regulating endopeptidase homolog, X-linked
RGD1559536	ENSRNOG00000039250	498855	X	similar to vitellogenin-like 1 precursor
Ar	ENSRNOG00000005639	24208	X	androgen receptor
Tsx	ENSRNOG00000002925	29391	X	testis specific X-linked gene
Tbx22	ENSRNOG00000002346	302369	X	T-box transcription factor 22
Srpx2	ENSRNOG00000003715	317181	X	sushi-repeat-containing protein, X-linked 2
Armcx2	ENSRNOG00000025705	367903	X	armadillo repeat containing, X-linked 2
Cldn2	ENSRNOG00000054495	300920	X	claudin 2
Dock11	ENSRNOG00000013321	313438	X	dedicator of cytokinesis 11
Zdhhc9	ENSRNOG00000004581	302808	X	zinc finger, DHHC-type containing 9
Zcchc18	ENSRNOG00000055939	679126	X	zinc finger, CCHC domain containing 18
LOC103694556	ENSRNOG00000052020	1.04E+08	Y	ubiquitin-like modifier-activating enzyme 1 Y

Table S7: Summary of gene ontology of up regulated genes in the testis of rats exposed to perfluorooctanoic acid

**S.No**	**Gene ontology term: Biological process**	**No. of genes**	**%#**
1	cellular process (GO:0009987)	22	25.90%
2	localization (GO:0051179)	2	2.40%
3	biological process involved in interspecies interaction between organisms (GO:0044419)	3	3.50%
4	biological regulation (GO:0065007)	15	17.60%
5	response to stimulus (GO:0050896)	11	12.90%
6	signaling (GO:0023052)	8	9.40%
7	developmental process (GO:0032502)	2	2.40%
8	multicellular organismal process (GO:0032501)	2	2.40%
9	biological adhesion (GO:0022610)	1	1.20%
10	locomotion (GO:0040011)	1	1.20%
11	metabolic process (GO:0008152)	12	14.10%
12	immune system process (GO:0002376)	6	7.10%
**S.No**	**Gene ontology term: Molecular Function**	**No. of genes**	**%#**
1	transcription regulator activity (GO:0140110)	1	2.80%
2	molecular transducer activity (GO:0060089)	4	11.10%
3	binding (GO:0005488)	13	36.10%
4	molecular function regulator (GO:0098772)	3	8.30%
5	catalytic activity (GO:0003824)	13	36.10%
6	transporter activity (GO:0005215)	2	5.60%
**S.No**	**Gene ontology term: Cellular component**	**No. of genes**	**%#**
1	cellular anatomical entity (GO:0110165)	23	88.50%
2	protein-containing complex (GO:0032991)	3	11.50%

# indicates percent calculation against total number of genes under gene ontology term

Table S8: KEGG pathway analysis of up regulated genes in the testis of rats exposed to perfluorooctanoic acid during prepubertal period

Term	Count	%	P Value	Fold Enrichment
rno04215:Apoptosis - multiple species	6	14.28571	1.65E-08	67.46324
rno05134:Legionellosis	5	11.90476	1.16E-05	32.95618
rno01524:Platinum drug resistance	6	14.28571	1.59E-06	27.63554
rno04115:p53 signaling pathway	5	11.90476	3.24E-05	25.48611
rno04623:Cytosolic DNA-sensing pathway	4	9.52381	5.94E-04	22.82338
rno04210:Apoptosis	7	16.66667	7.79E-07	19.67678
rno04668:TNF signaling pathway	5	11.90476	1.61E-04	16.91556
rno05222:Small cell lung cancer	4	9.52381	0.001494	16.62138
rno05162:Measles	6	14.28571	2.38E-05	15.92882
rno05133:Pertussis	3	7.142857	0.014181	15.71062
rno05142:Chagas disease	4	9.52381	0.002125	14.70353
rno05161:Hepatitis B	6	14.28571	3.61E-05	14.60987
rno05160:Hepatitis C	6	14.28571	3.72E-05	14.51741
rno04936:Alcoholic liver disease	5	11.90476	3.19E-04	14.15895
rno05164:Influenza A	6	14.28571	5.14E-05	13.57249
rno04512:ECM-receptor interaction	3	7.142857	0.020192	13.03267
rno04657:IL-17 signaling pathway	3	7.142857	0.021947	12.46603
rno04620:Toll-like receptor signaling pathway	3	7.142857	0.022396	12.33199
rno04932:Non-alcoholic fatty liver disease	5	11.90476	6.23E-04	11.87241
rno05169:Epstein-Barr virus infection	7	16.66667	1.49E-05	11.84089
rno04650:Natural killer cell mediated cytotoxicity	3	7.142857	0.025641	11.46875
rno04933:AGE-RAGE signaling pathway in diabetic complications	3	7.142857	0.02612	11.3552
rno05417:Lipid and atherosclerosis	6	14.28571	1.41E-04	10.97488
rno05152:Tuberculosis	5	11.90476	8.90E-04	10.7992
rno04217:Necroptosis	4	9.52381	0.007509	9.381391
rno05167:Kaposi sarcoma	5	11.90476	0.001861	8.849344
rno05170:Human immunodeficiency virus 1 infection	5	11.90476	0.002493	8.168625
rno05163:Human cytomegalovirus infection	5	11.90476	0.003077	7.707493
rno05165:Human papillomavirus infection	7	16.66667	1.80E-04	7.580855
rno05132:Salmonella infection	5	11.90476	0.003354	7.525427
rno05203:Viral carcinogenesis	4	9.52381	0.017112	6.919306
rno05200:Pathways in cancer	9	21.42857	2.97E-05	6.395214
rno05168:Herpes simplex virus 1 infection	6	14.28571	0.002306	5.942358
rno05010:Alzheimer disease	6	14.28571	0.00244	5.866368
rno05022:Pathways of neurodegeneration - multiple diseases	7	16.66667	9.05E-04	5.61015
rno05020:Prion disease	4	9.52381	0.029721	5.601343
rno05016:Huntington disease	4	9.52381	0.040048	4.980999

Table S9: Summary of gene ontology of down regulated genes in the testis of rats exposed to perfluorooctanoic acid

**S.No**	**Gene ontology term: Biological process**	**No. of genes**	**%#**
1	cellular process (GO:0009987)	26	23.40%
2	reproductive process (GO:0022414)	2	1.80%
3	localization (GO:0051179)	7	6.30%
4	reproduction (GO:0000003)	2	1.80%
5	biological regulation (GO:0065007)	19	17.10%
6	response to stimulus (GO:0050896)	13	11.70%
7	signaling (GO:0023052)	10	9.00%
8	developmental process (GO:0032502)	7	6.30%
9	rhythmic process (GO:0048511)	1	0.90%
10	biological adhesion (GO:0022610)	2	1.80%
11	locomotion (GO:0040011)	1	0.90%
12	multicellular organismal process (GO:0032501)	6	5.40%
13	metabolic process (GO:0008152)	15	13.50%
**S.No**	**Gene ontology term: Molecular Function**	**No. of genes**	**%#**
1	transcription regulator activity (GO:0140110)	6	11.50%
2	molecular transducer activity (GO:0060089)	6	11.50%
3	molecular adaptor activity (GO:0060090)	1	1.90%
4	binding (GO:0005488)	19	36.50%
5	molecular function regulator (GO:0098772)	2	3.80%
6	catalytic activity (GO:0003824)	14	26.90%
7	transporter activity (GO:0005215)	4	7.70%
**S.No**	**Gene ontology term: Cellular component**	**No. of genes**	**%#**
1	cellular anatomical entity (GO:0110165)	23	85.20%
2	protein-containing complex (GO:0032991)	4	14.80%

# indicates percent calculation against total number of genes under gene ontology term

Table S10: KEGG pathway analysis of down regulated genes in the testis of rats exposed to perfluorooctanoic acid during prepubertal period

Term	Count	%	P Value	Fold Enrichment
rno00480:Glutathione metabolism	4	8.695652	0.001779	15.92882
rno04913:Ovarian steroidogenesis	3	6.521739	0.016238	14.83028
rno04918:Thyroid hormone synthesis	3	6.521739	0.025021	11.78296
rno04512:ECM-receptor interaction	3	6.521739	0.035328	9.774503
rno04080:Neuroactive ligand-receptor interaction	5	10.86957	0.040866	3.675881
rno01100:Metabolic pathways	10	21.73913	0.078884	1.784186

Table S11: Summary of gene ontology of up regulated genes in the epididymis of rats exposed to perfluorooctanoic acid

**S.No**	**Gene ontology term: Biological process**	**No. of genes**	**%#**
1	cellular process (GO:0009987)	61	28.00%
2	reproductive process (GO:0022414)	3	1.40%
3	localization (GO:0051179)	22	10.10%
4	biological process involved in interspecies interaction between organisms (GO:0044419)	2	0.90%
5	reproduction (GO:0000003)	3	1.40%
6	biological regulation (GO:0065007)	31	14.20%
7	response to stimulus (GO:0050896)	19	8.70%
8	signaling (GO:0023052)	14	6.40%
9	developmental process (GO:0032502)	10	4.60%
10	multicellular organismal process (GO:0032501)	13	6.00%
11	biological adhesion (GO:0022610)	3	1.40%
12	metabolic process (GO:0008152)	32	14.70%
13	immune system process (GO:0002376)	5	2.30%
**S.No**	**Gene ontology term: Molecular Function**	**No. of genes**	**%#**
1	transcription regulator activity (GO:0140110)	5	4.80%
2	molecular transducer activity (GO:0060089)	6	5.80%
3	binding (GO:0005488)	40	38.50%
4	structural molecule activity (GO:0005198)	2	1.90%
5	molecular function regulator (GO:0098772)	7	6.70%
6	catalytic activity (GO:0003824)	34	32.70%
7	transporter activity (GO:0005215)	10	9.60%
**S.No**	**Gene ontology term: Cellular component**	**No. of genes**	**%#**
1	cellular anatomical entity (GO:0110165)	77	90.60%
2	protein-containing complex (GO:0032991)	8	9.40%

# indicates percent calculation against total number of genes under gene ontology term

Table S12: KEGG pathway analysis of up regulated genes in the epididiymis of rats exposed to perfluorooctanoic acid during prepubertal period

Term	Count	%	PValue	Fold Enrichment
rno00480:Glutathione metabolism	3	1.923077	0.048064	6.371528
rno01100:Metabolic pathways	20	12.82051	0.004359	1.903132
rno00980:Metabolism of xenobiotics by cytochrome P450	3	1.923077	0.085657	6.036184
rno04721:Synaptic vesicle cycle	3	1.923077	0.089534	5.88141

Table S13: Summary of gene ontology of down regulated in the epididymis of rats exposed to perfluorooctanoic acid during prepubertal period

**S.No**	**Gene ontology term: Biological process**	**No. of genes**	**%#**
1	developmental process (GO:0032502)	48	5.40%
2	multicellular organismal process (GO:0032501)	52	5.80%
3	cellular process (GO:0009987)	234	26.20%
4	reproduction (GO:0000003)	6	0.70%
5	localization (GO:0051179)	64	7.20%
6	reproductive process (GO:0022414)	6	0.70%
7	biological adhesion (GO:0022610)	14	1.60%
8	immune system process (GO:0002376)	9	1.00%
9	biological regulation (GO:0065007)	143	16.00%
10	growth (GO:0040007)	7	0.80%
11	signaling (GO:0023052)	80	8.90%
12	metabolic process (GO:0008152)	117	13.10%
13	biological process involved in interspecies interaction between organisms (GO:0044419)	10	1.10%
14	response to stimulus (GO:0050896)	88	9.80%
15	biological phase (GO:0044848)	1	0.10%
16	behavior (GO:0007610)	1	0.10%
17	rhythmic process (GO:0048511)	1	0.10%
18	locomotion (GO:0040011)	13	1.50%
**S.No**	**Gene ontology term: Molecular Function**	**No. of genes**	**%#**
1	transporter activity (GO:0005215)	39	10.50%
2	transcription regulator activity (GO:0140110)	23	6.20%
3	catalytic activity (GO:0003824)	109	29.20%
4	cytoskeletal motor activity (GO:0003774)	4	1.10%
5	molecular function regulator (GO:0098772)	29	7.80%
6	ATP-dependent activity (GO:0140657)	9	2.40%
7	molecular transducer activity (GO:0060089)	39	10.50%
8	molecular adaptor activity (GO:0060090)	2	0.50%
9	structural molecule activity (GO:0005198)	1	0.30%
10	binding (GO:0005488)	118	31.60%
**S.No**	**Gene ontology term: Cellular component**	**No. of genes**	**%#**
1	cellular anatomical entity (GO:0110165)	270	87.90%
2	protein-containing complex (GO:0032991)	37	12.10%

# indicates percent calculation against total number of genes under gene ontology term

Table S14: KEGG pathway analysis of down regulated genes in the epididiymis of rats exposed to perfluorooctanoic acid during prepubertal period

Term	Count	%	PValue	Fold Enrichment
rno04310:Wnt signaling pathway	14	3.301887	7.52E-05	3.800296
rno04020:Calcium signaling pathway	17	4.009434	1.09E-04	3.107072
rno04976:Bile secretion	9	2.122642	9.61E-04	4.392287
rno05224:Breast cancer	10	2.358491	0.004855	3.099662
rno04713:Circadian entrainment	8	1.886792	0.005324	3.744898
rno04261:Adrenergic signaling in cardiomyocytes	10	2.358491	0.005771	3.018092
rno04514:Cell adhesion molecules	11	2.59434	0.005779	2.803472
rno04360:Axon guidance	11	2.59434	0.006016	2.787983
rno05200:Pathways in cancer	22	5.188679	0.006346	1.875929
rno04921:Oxytocin signaling pathway	10	2.358491	0.006542	2.959677
rno04934:Cushing syndrome	10	2.358491	0.00739	2.903481
rno04972:Pancreatic secretion	8	1.886792	0.007719	3.495238
rno04970:Salivary secretion	7	1.650943	0.008628	3.916159
rno04080:Neuroactive ligand-receptor interaction	17	4.009434	0.010652	1.999679
rno05022:Pathways of neurodegeneration - multiple diseases	19	4.481132	0.015465	1.827306
rno05031:Amphetamine addiction	6	1.415094	0.015782	4.047794
rno04916:Melanogenesis	7	1.650943	0.02045	3.243687
rno04024:cAMP signaling pathway	11	2.59434	0.021845	2.283371
rno04010:MAPK signaling pathway	13	3.066038	0.031466	1.974752
rno04911:Insulin secretion	6	1.415094	0.037099	3.238235

Table S15: Differentially expressed genes in the testis and epididymis of rats exposed to perfluorooctanoic acid during prepubertal period

Gene symbol	Fold change (RNA-Seq)#	Fold Change (qPCR)##
Steroidogenic pathway$		
hsd17β3	-2.18	-2.52
StAR	-2.49	-1.84
Androgen signalling$		
AR	-1.54$	-2.04$
	-2.24@	-1.97@
Apoptosis$		
Casp3	3.79	2.67
Antioxidant system$		
Nfe2l2	-2.41	-2.07
Receptors associated with leydig cells$		
Lhcgr	-2.39	-2.79
Neuroactive signaling pathway@		
Alpha adrenoceptor 1	-3.79	-3.18
Muscarinic acetylcholine receptor 3	-4.22	-2.64

#Fold change refers to gene expression in the testis and epididymis of PFOA exposed rats as compared to untreated controls using RNA-Seq analysis (sample size: 03)
##Fold change refers to gene expression in the testis and epididymis of PFOA exposed rats as compared to untreated controls using qPCR (sample size: 03)
Only mean values were shown; $performed in testis; @performed in epididymis.
